# Differential metabolic responses of shrubs and grasses to water additions in arid karst region, southwestern China

**DOI:** 10.1038/s41598-019-46083-1

**Published:** 2019-07-03

**Authors:** Muhammad Umair, Ningxiao Sun, Hongmei Du, Jun Yuan, Arshad Mehmood Abbasi, Jiahao Wen, Wenjuan Yu, Jinxing Zhou, Chunjiang Liu

**Affiliations:** 1 School of Agriculture and Biology, Shanghai Jiao Tong University, Shanghai, 200240 China; 2 Design School, Shanghai Jiao Tong University, Shanghai, 200240 China; 3 Department of Environment Sciences, COMSATS University, Islamabad, Abbottabad Campus Pakistan; 4 Instrumental Analysis Center, Shanghai Jiao Tong University, Shanghai, China; 5 Yunnan Karst Ecosystem Research Station, School of Water and Soil Conservation, Beijing Forestry University, Beijing, China; 6 Shanghai Urban Forest Research Station, State Forestry Administration, Shanghai, China; 7 Key Laboratory of Urban Agriculture (South), Ministry of Agriculture, Shanghai, 22060 China

**Keywords:** Climate-change ecology, Climate-change impacts

## Abstract

Increasing precipitation has been predicted to occur in the karst areas in southwestern regions of China. However, it is little known how various plants respond to increasing precipitation in this region. Here we determined the impacts of water addition on leaf metabolites of grasses (*Cymbopogon distans and Arundinella sitosa*) and shrubs (*Carissa spinarum* and *Bauhinia brachycarpa*) in this area. Four levels of water additions (CK, T1, T2 and T3 indicating 0%, +20%, +40% and +60% relative to the current monthly precipitation, respectively) were designed. Sphingolipids substantially increased in the leaves of all four species with increasing water supply which suggests that these plants adopted biochemical strategy to tolerate the wet stress. However, both shrubs showed decreases in valine and threonine (amino acids), threonate, succinate and ascorbic acid (organic acids), galactose and rhamnose (sugars) and epicatchin and oleamides (secondary metabolites) with increasing water supply. Both grasses increased in the total metabolites at T1, but the total metabolites in *A*. *sitosa* significantly decreased at T2 and T3 while remains unchanged in *C*. *distans*. Tri-carboxylic acid cycle and amino acid metabolism in shrubs and shikimate pathway in grasses were strongly affected with water supply. Overall, shrubs and grasses respond differentially to variation in water addition in terms of metabolomics, which is helpful in understanding how plants respond to climate change.

## Introduction

Drought and soil erosion are the major causes of desertification, which lead to extensive degradation of land and decline of vegetation in karst area. Less water retention capacity of karst is characterized by an extremely slow soil formation from underlying limestone in this region^1–3^. Therefore, drought is one of the most important factor in limiting the growth, photosynthesis and distribution of plants in the karst habitats of southwestern China^1,4^. It has been reported that drought stress not only reduces stem elongation, root propagation and leaf size but also disturbs plant-water relations and reduces water-use efficiency in plants^5,6^. As a result, the aboveground biomass of forest and vegetation in tropical and subtropical zones of the karst areas is approximately equal to that of temperate zone^7^. Moreover, majority of the plant species growing in karst have adapted to the conditions of arid climate in long-term evolution. Hence, increasing water supply may cause an oxidative stress in plant species growing in karst area^8^. Due to decrease in the number of rain days/annum, an increase in annual precipitation has been observed in southwest China for the last decade^9,10^. However, the karst habitat supports plant species in adaptation but the plants responses to increasing water remains mysterious.

Metabolomics contributes significantly in expanding our knowledge on metabolic changes and biochemical composition of plant species growing in diverse environmental conditions^11,12^. Eco-metabolomics deals with metabolome and metabolic remodeling, which are down and up-regulated due to environmental stresses. Furthermore, Eco-metabolomics evaluates the effects of the plant–environment interactions using advanced techniques such as GC-MS and LCMS coupled with advanced bio-informatics tools^12,13^. Additionally, eco-metabolomics approach is progressively being utilized to observe the modification in a specific genotype due to changes in global environment, especially the drought stress^14–16^. Differential responses of grasses and shrubs to climatic variability, particularly increasing precipitation inconsistency, have been reported mainly on their average productivity. It has been estimated that precipitation variability significantly reduces the primary production of an ecosystem^17^.

Water is an important factor for proper functioning and restoration of ecosystems in desert and grassland regions^17,18^. Changes in the hydrological systems not only effect the rate of diffusion of plant nutrients but also alter the vegetation pattern^19,20^. Moreover, changes in the components of hydrological systems, such as rainfall, impact significantly on the current water gradient of habitat and plant communities^21,22^. Other components include surface water^23,24^, ground water^25,26^ and soil moisture^27,28^. Over a long period of drought, decrease in nutrient uptake is accounted by partial transfer of ions to the root system^29^. Therefore, field manipulative experiments of irrigation are the best way to explore the relationships between water availability and ecosystem functioning^17,30^. In addition, maximum benefits may be attained by adopting suitable irrigation and planting techniques under water stress condition^31^.

In southwestern China, major part of the plant community in karst areas is dominated by drought-resistant dwarf shrubs and grasses. These species have distinct water relations with variable water conditions and have physiological and biochemical adaptations to survive in the arid climate of karst for long time. However, increasing precipitation may cause a wet stress in these species, particularly growing in the karst region^8^. In addition, the response of different plant species to increasing precipitation in the arid climate of karst area in terms of metabolomics is still unresolved. The main contribution of this work is (i) to determine whether plants respond differently to watering treatments in karst areas of Yunnan province, SW China (ii) to examine the impacts of water addition on the leaf metabolites of four plants species.

## Results

### Metabolic responses of plants to water additions

**Figure 1:**
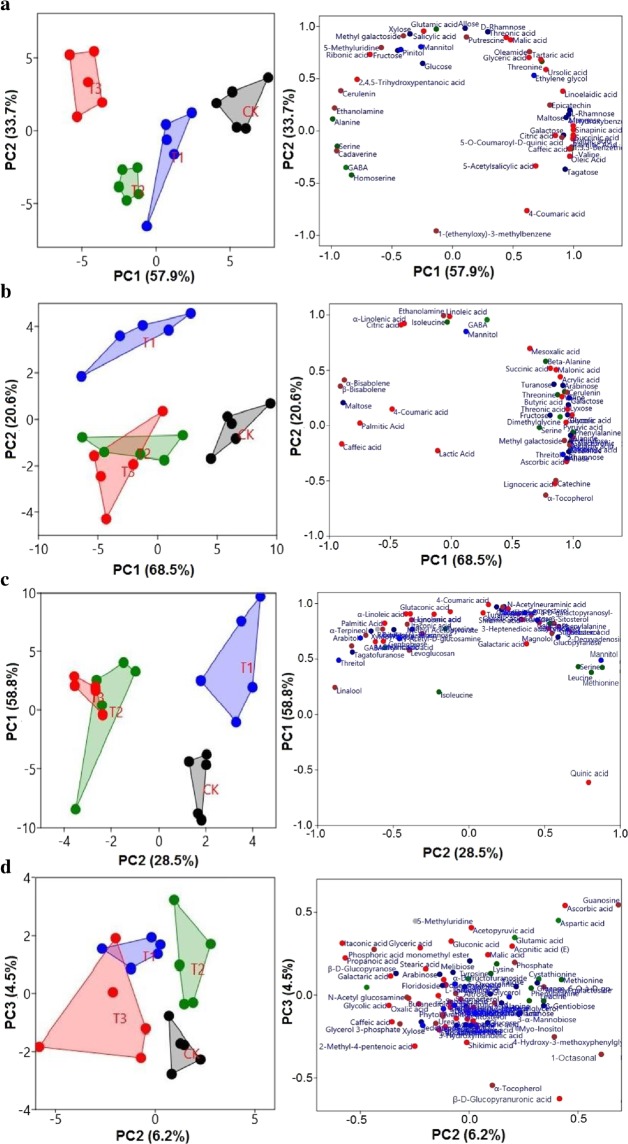
Plot of significant metabolomic variables in the PCA of four plant species (**a**) *C*. *spinarum* (**b**) *B*. *brachycarpa* (**c**) *C*. *distans* and (**d**) *A*. *sitosa* under different levels of watering treatments (*p* < 0.05). Water treated samples are indicated by different colors and geometrical figures. Loadings of metabolomic variables in PC1 and PC2 are shown. The various metabolites groups are represented by colors: dark blue, sugars; dark green, amino acids; green, amino-acid derivatives; red, organic acids; dark gray, nucleotides; and brown, terpenes and phenolics.

Results given in Fig. 1 revealed that the overall leaf metabolism was significantly affected by water addition with significant difference at *p* < 0.05. The PCA results obtained from the data for four plant species indicate significant differences in leaf metabolites, which is mainly depending on the levels of watering treatments. PCA of metabolomics variables of shrubs exhibited that samples had different values along the PC2-axis. However, significant changes were observed between CK and water treated samples along the PC1-axis at *p* < 0.0001 (Fig. 1a,b). *Post hoc* analysis of the PCA scores exposed that overall leaf metabolism of *C*. *spinarum* varied significantly between CK and T3 along the first PC (explaining 57.9% of the variation, *p* < 0.0001). Data of metabolomics variables, given in Fig. 1a, (PC1) showed that water treated samples had higher concentrations of amino acids (compounds associated with protein biosynthesis), secondary metabolites and sugar alcohol while the CK samples had higher concentrations of sugars, organic acids and osmolytes. PCA of metabolomics variables of *B*. *brachycarpa* (Fig. 1b) depicted significant changes between CK and T3 which were separated along the PC1 axis explaining 68.5% of the variation (*p* < 0.01). These results were confirmed by *Post hoc* analysis. PC1 loadings of metabolomics variables showed that CK samples had higher concentrations of organic acids i.e., intermediates of TCA cycle and amino acids while water treated samples had higher concentrations of fatty acids and osmolytes (Fig. 1b).

In contrast, the PCA of metabolomics variables of grasses exhibited that CK and water treated samples were separated along the PC2-axis (Fig. 1c,d). PCA explaining 87.3% of the variation with metabolomics variables of *C*. *distans* showed that CK and water treated samples differed along the PC2-axis (Fig. 1c). *Post hoc* analysis of the PC2 scores specified that overall leaf metabolism of *C*. *distans* varied considerably between CK and T3 (*p* < 0.01). Loadings of metabolomics variables in PC2 disclosed that water treated samples had higher concentrations of fatty acids and sugars related compounds while CK group had higher concentrations of amino acids, osmolytes and organic acids, like the intermediates in Shikimate pathway (Fig. 1c). Likewise, PCA of metabolomics variables of *A*. *sitosa* indicated comparable responses to water addition which exposed that CK and T3 were separated along the PC2-axis (explaining 6.4% of the variation, *p* < 0.01). PC2 and PC3 elucidated 10% of the variance in the PCA conducted with the leaf samples of *A*. *sitosa*. PC1 was discounted for the differences of watering samples and results were confirmed by *Post hoc* analysis. PC2 loadings of metabolomics variables indicate that water treated samples had lower concentrations of amino acids (Fig. 1d).

### Metabolic regulations during watering treatments

**Figure 2:**
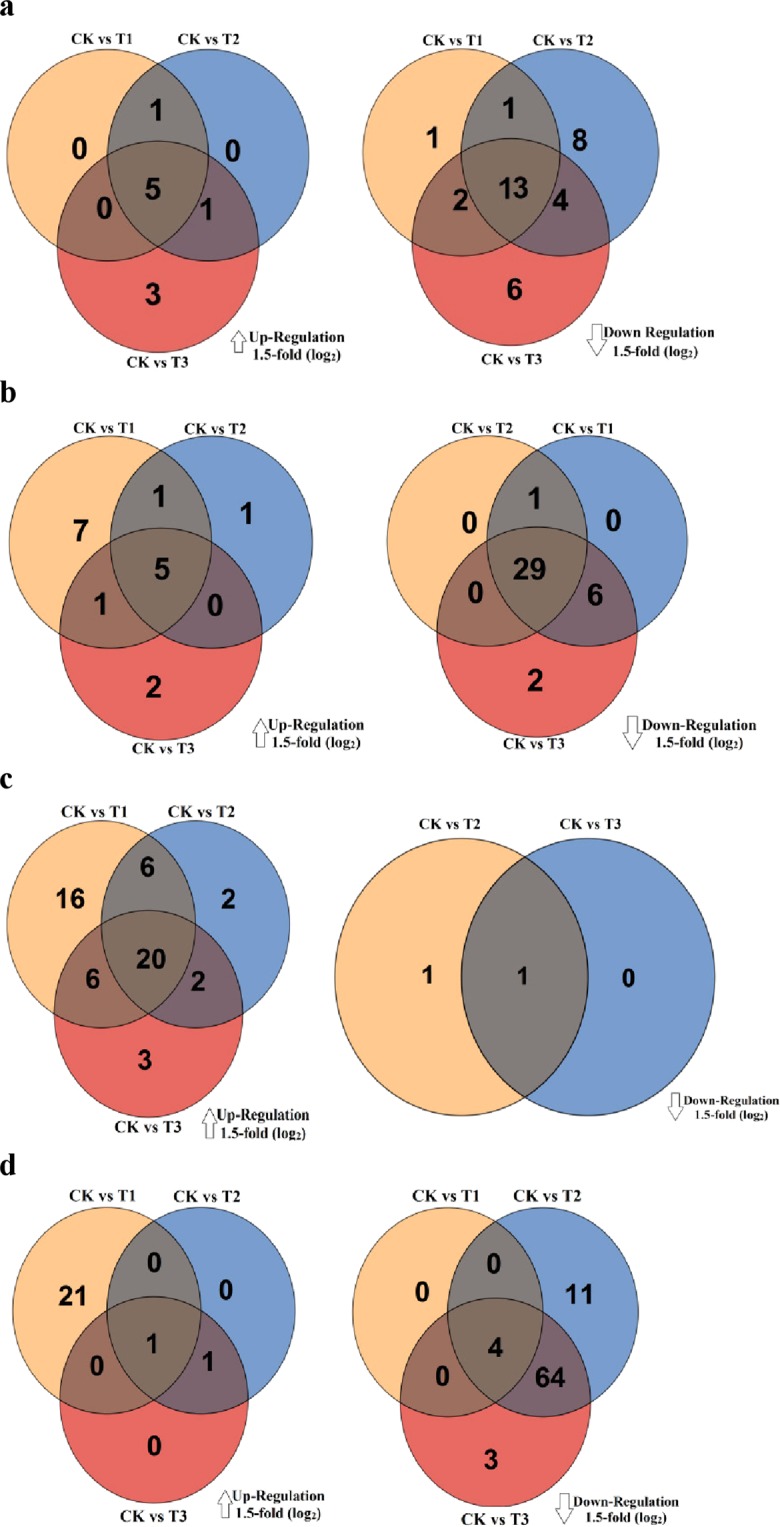
Venn diagram illustrating differential regulating metabolites in four plant species (**a**) *C*. *spinarum* (**b**) *B*. *brachycarpa* (**c**) *C*. *distans* and (**d**) *A*. *sitosa* under different levels of watering treatments.

The GC/MS chromatogram given in Fig. S1 indicates that more than 450 peaks were resolved from the polar extract of each plant species. Comparatively, more metabolites were identified in the leaves of grass species (181 metabolites) than shrubs (152 metabolites). In *C*. *distans* and *A*. *sitosa* leaves, there were 141 and 136 metabolites, respectively while 108 and 110 metabolites were found in *C*. *spinarum* and *B*. *brachycarpa* leaves (Tables S1–S4). The numbers of unique and common leaf metabolites that increased or decreased during watering treatment are presented in Venn diagrams (Fig. 2a–d).

**Figure 3:**
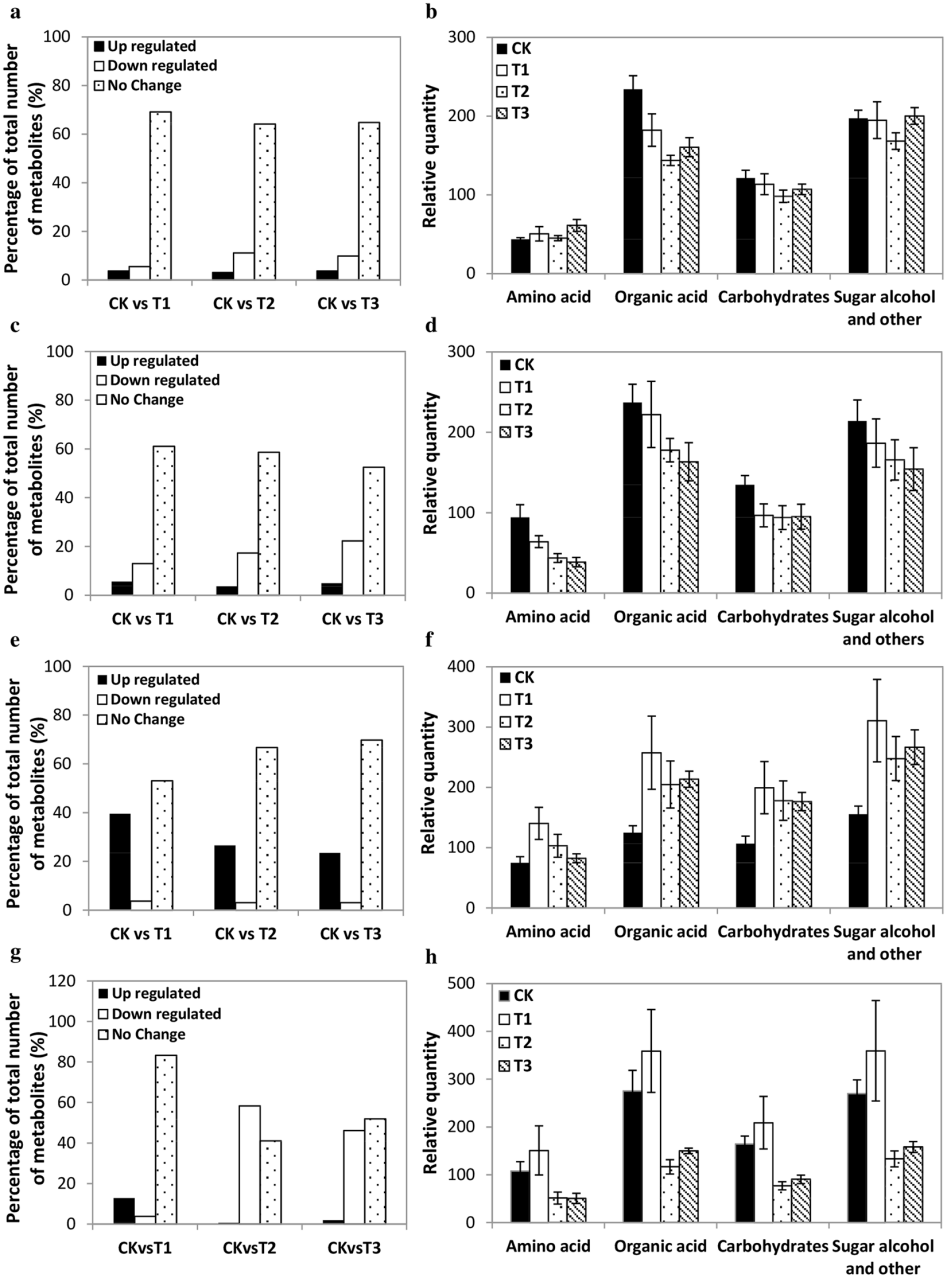
Changes to the percentage of total number of metabolites (%) in the leaves of four plant species (**a**) *C*. *spinarum* (**c**) *B*. *brachycarpa* (**e**) *C*. *distans* and (**g**) *A*. *sitosa* under different levels of watering treatments, and total relative amino acid, organic acid, sugar and sugar alcohol content in four plant species (**b**) *C*. *spinarum* (**d**) *B*. *brachycarpa* (**f**) *C*. *distans* and (**h**) *A*. *sitosa*. Vertical bars above columns indicate standard error (±SE) of each mean.

Regulation of the significant leaf metabolites among the four treatment groups i.e. CK, T1, T2 and T3 (cutoff of 2-fold log2, *p* < 0.05) was compared. This analysis revealed that five metabolites in *C*. *spinarum* and *B*. *brachycarpa* leaves each, 20 in *C*. *distans* leaves and one in *A*. *sitosa* leaves were consistently up-regulated at T1, T2 and T3 (*p* < 0.05). Compared to CK, the down-regulated metabolites in the leaves of both shrubs were significantly higher at T3 (*p* < 0.05). The down-regulated metabolites in *A*. *sitosa* leaves were found much higher than *C*. *distans* leaves at T2 and T3, while only one metabolite in *C*. *distans* leaves was consistently down-regulated at T2 and T3 (Fig. 2c,d). The percentage of total number of down-regulated metabolites in the leaves of both shrubs was higher than up-regulated metabolites (Fig. 3a,c,e,g), whereas the percentage of down-regulated metabolites was 11.1% in *C*. *spinarum* leaves at T2 and 22.2% in *B*. *brachycarpa* leaves at T3 (Fig. 3a,c). Comparatively, the percentage of up-regulated metabolites in both *C*. *distans* and *A*. *sitosa* leaves was 53.1% and 12.8%, respectively at T1 (Fig. 3e,g). However, the percentage of down-regulated metabolites in *A*. *sitosa* leaves was 58.3% in T2 and 46.2% at T3 compared to CK (Fig. 3g).

It was noted that total relative content of metabolites decreased in the leaves of both shrubs with water additions (Fig. 3b,d). Compared to CK, the total content of organic acids in *C*. *spinarum* and *B*. *brachycarpa* leaves was decreased by 1.20 and 0.71 folds at T2, and 0.93 and 0.92 folds at T3 (*p* < 0.05), respectively. Conversely, in *C*. *distans* and *A*. *sitosa* leaves, amino acids, organic acid, carbohydrates, sugar alcohols and other metabolites contents increased at T1, but were lower than CK at T2 and T3 in *A*. *sitosa* (Fig. 3f,h). For instance, the total relative content of amino acids, organic acids, carbohydrates and sugar alcohols, and other metabolites in *C*. *distans* leaves increased by 1.55, 1.79, 1.55 and 1.71 folds, respectively, at T1 compared to CK. Whereas, compared to CK, the total content of amino acids, carbohydrates, organic acids, and sugar alcohols, and other metabolites in *A*. *sitosa* leaves increased by 0.82, 0.65, 0.59 and 0.70 folds at T1, but dropped up to 1.85, 2.12, 1.88 and 1.75 folds, respectively.

### Variation in different metabolites due to water addition

**Figure 4:**
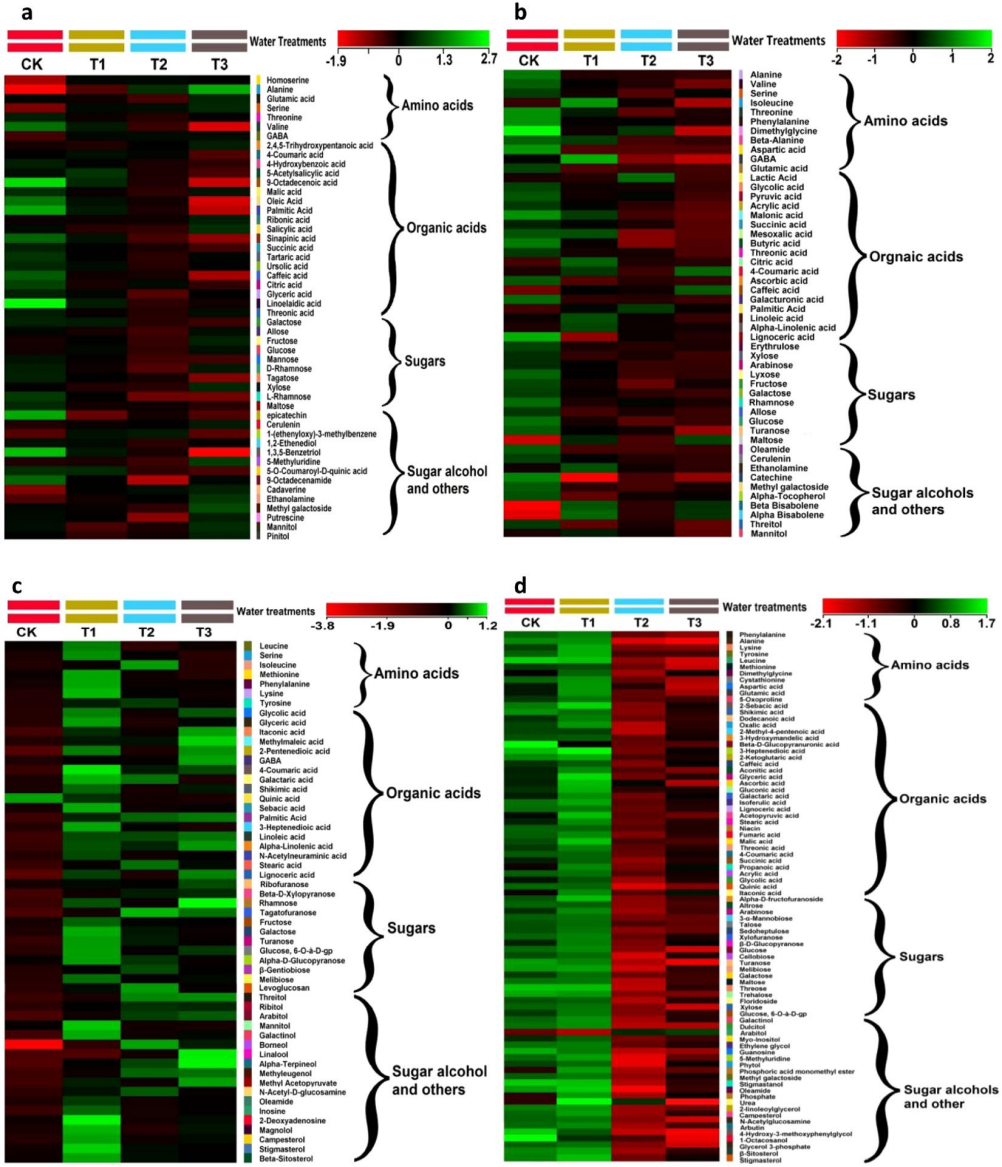
Heat maps of significant changes in metabolites groups of four plant species (**a**) *C*. *spinarum* (**b**) *B*. *brachycarpa* (**c**) *C*. *distans* and (**d**) *A*. *sitosa* in response to water addition (*P* < 0.05). Each colored cell on the map corresponds to a normalized log response value of the metabolite levels, with samples in columns and metabolites in rows. Green and red colors indicate increased and decreased metabolite concentrations, respectively.

It was observed that water additions had significant effects on the amino acid content in the leaves of both shrubs and grasses (Fig. 4a–d). Compared to CK, threonine and valine content was decreased significantly at T1, T2 and T3 in the leaves of both shrubs (Fig. 4a,b). However, the content of GABA and homoserine increased considerably at T1, T2 and T3 in *C*. *spinarum* leaves (*p* < 0.05). Compared to the CK, the relative content of alanine and serine increased by 3.55 and 1.32 folds, respectively, in *C*. *spinarum* leaves at T3 (*p* < 0.05) (Table 1). However, other amino acids did not show any significant change after water addition. In *B*. *brachycarpa* leaves, a decline was noted in the content of amino acids, except GABA and isoleucine at T1, T2 and T3 (Table 2). As shown in Table 3, water addition led to an increase in the relative content of eight amino acids in *C*. *distans* leaves, such as GABA, isoleucine, leucine, lysine, serine, methionine, phenylalanine and tyrosine at T1 (*p* < 0.05). Compared to CK, the relative content of glutamic acid in *A*. *sitosa* leaves (Table 4) increased by 1.07 fold at T1 (*p* < 0.05), but at T2 and T3, the relative content of cystathionine, oxoproline, methionine, phenylalanine and leucine decreased significantly (*p* < 0.05).

**Table 1:** Relative concentrations and fold changes of significant metabolites in the leaves of *C*. *spinarum* under different levels of watering treatments.

Metabolite groups	RT	Metabolites	Relative concentration				Fold changes			*p*
			CK	T1	T2	T3	FC^T1/CK^	FC^T2/CK^	FC^T3/CK^	
**Amino acids**	10.17	Alanine	1.06 ± 0.45	2.41 ± 1.15	4.08 ± 0.72	12.45 ± 3.24	1.18	1.94	3.55*	0.002
	21.43	GABA	3.52 ± 0.44	5.43 ± 0.66	5.46 ± 0.49	5.59 ± 0.69	0.63*	0.64*	0.67*	0.046
	22.58	Glutamic acid	5.14 ± 0.59	4.73 ± 0.86	3.37 ± 0.39	6.77 ± 1.05	−0.12	−0.61	0.40	0.046
	26.73	Homoserine	2.76 ± 0.79	5.94 ± 0.88	5.82 ± 0.48	5.48 ± 0.39	1.10*	1.07*	0.99*	0.006
	14.48	Serine	2.72 ± 0.60	5.14 ± 0.69	5.31 ± 0.71	6.82 ± 1.23	0.92	0.96	1.32*	0.010
	21.95	Threonine	6.36 ± 0.40	4.80 ± 0.75	4.04 ± 0.32	4.80 ± 0.24	−0.41*	−0.65*	−0.41*	0.029
	32.54	Valine	9.47 ± 1.93	5.55 ± 1.59	3.35 ± 1.02	1.63 ± 0.56	−0.77	−1.50*	−2.54*	0.009
**Organic acids**	24.23	2,4,5-Trihydroxy pentanoic acid	4.46 ± 0.43	4.20 ± 0.49	4.96 ± 0.36	6.38 ± 0.55	−0.09	0.15	0.52*	0.03
	27.32	4-Coumaric acid	5.27 ± 0.63	5.79 ± 0.31	5.41 ± 0.37	3.54 ± 0.48	0.14	0.04	−0.57*	0.023
	23.75	4-Hydroxybenzoic acid	6.37 ± 0.13	5.24 ± 0.59	4.30 ± 0.33	4.09 ± 0.80	−0.28	−0.57*	−0.64*	0.036
	38.02	5-Acetylsalicylic acid	5.34 ± 0.59	7.29 ± 1.01	4.16 ± 0.60	3.21 ± 0.25	0.45	−0.36	−0.73	0.002
	30.48	Ascorbic acid	5.77 ± 0.52	4.27 ± 1.53	3.02 ± 0.76	6.95 ± 0.71	−0.74	−1.60	0.46	0.050
	34.11	Caffeic acid	9.49 ± 1.02	4.35 ± 0.57	4.12 ± 0.35	2.04 ± 0.33	−1.13*	−1.20*	−2.21**	2.58E-06
	27.93	Citric acid	7.73 ± 1.21	3.91 ± 0.52	4.89 ± 0.55	3.47 ± 0.49	−0.98*	−0.66*	−1.16*	0.012
	35.57	Elaidic acid	14.07 ± 5.69	3.21 ± 0.51	1.89 ± 0.21	0.82 ± 0.10	−2.13*	−2.89*	−4.1*	1.99E-06
	29.36	Glyceric acid	6.47 ± 0.67	5.55 ± 1.04	3.19 ± 0.30	4.79 ± 0.24	−0.22	−1.02*	−0.43	0.013
	35.44	Linoelaidic acid	14.86 ± 6.97	2.12 ± 0.30	1.27 ± 0.08	1.74 ± 0.60	−2.81*	−3.55*	−3.09*	1.12E-04
	14.63	Malic acid	6.56 ± 0.71	4.25 ± 0.27	3.61 ± 0.42	5.57 ± 0.94	−0.63*	−0.86**	−0.24	0.015
	35.69	Oleic Acid	10.24 ± 2.13	4.69 ± 0.66	3.68 ± 0.37	1.38 ± 0.13	−1.13*	−1.48*	−2.89*	3.98E-07
	32.49	Palmitic Acid	12.43 ± 4.54	3.79 ± 0.53	2.58 ± 0.27	1.21 ± 0.16	−1.71*	−2.27*	−3.36*	7.20E-06
	37.72	Ribonic acid	4.54 ± 0.16	4.66 ± 0.54	4.51 ± 0.34	6.29 ± 0.50	0.04	−0.01	0.47*	0.042
	20.90	Salicylic acid	5.12 ± 0.39	3.93 ± 0.59	3.79 ± 0.67	7.17 ± 1.50	−0.38	−0.43	0.49	0.034
	45.29	Sinapic acid	9.26 ± 0.67	5.15 ± 0.83	3.06 ± 0.47	2.53 ± 0.96	−0.85*	−1.60*	−1.87**	4.14E-04
	23.32	Succinic acid	6.57 ± 0.54	5.42 ± 1.12	4.27 ± 0.19	3.74 ± 0.67	−0.28	−0.62	−0.82*	0.042
	23.07	Tartaric acid	6.88 ± 0.78	4.81 ± 0.89	3.53 ± 0.12	4.78 ± 0.52	−0.51*	−0.96*	−0.53	0.014
	21.76	Threonic acid	6.14 ± 0.76	4.56 ± 0.41	3.87 ± 0.17	5.44 ± 0.61	−0.43	−0.67*	−0.18	0.034
	57.83	Ursolic acid	6.37 ± 0.70	5.21 ± 0.69	3.67 ± 0.21	4.74 ± 0.64	−0.29	−0.79*	−0.43	0.050
**Sugar alcohols**	07.91	Ethylene glycol	5.73 ± 0.46	5.51 ± 0.49	3.95 ± 0.30	4.81 ± 0.38	−0.06	−0.54*	−0.25	0.031
	30.60	Myo-inositol	4.06 ± 0.47	4.38 ± 0.69	4.76 ± 0.63	6.80 ± 0.44	0.11	0.23	0.74**	0.015
	26.23	Mannitol	5.28 ± 0.32	3.22 ± 0.34	4.23 ± 0.56	7.26 ± 1.37	−0.71	−0.32	0.46	0.010
	27.85	Pinitol	5.01 ± 0.40	4.01 ± 0.75	4.48 ± 0.23	6.51 ± 0.58	−0.32	−0.16	0.38	0.022
**Sugars**	16.38	Allose	5.48 ± 0.15	4.83 ± 0.62	3.80 ± 0.32	5.88 ± 0.40	−0.18	−0.53*	0.10	0.013
	25.71	D-Rhamnose	6.44 ± 1.51	4.76 ± 1.01	3.05 ± 0.23	5.75 ± 0.57	−0.43	−1.08*	−0.16	0.043
	22.52	Fructose	4.49 ± 0.63	5.35 ± 1.05	3.73 ± 0.39	6.43 ± 0.19	0.25	−0.27	0.52	0.045
	36.52	Galactose	6.19 ± 0.27	6.39 ± 1.34	3.98 ± 0.65	3.44 ± 0.31	0.05	−0.63*	−0.85*	0.020
	29.87	Glucose	4.68 ± 0.43	5.58 ± 0.41	3.93 ± 0.25	5.82 ± 0.39	0.26	−0.25	0.31	0.007
	40.47	L-Rhamnose	8.18 ± 0.72	5.42 ± 0.96	3.22 ± 0.73	3.18 ± 0.72	−0.59*	−1.34*	−1.36*	0.008
	15.98	Maltose	6.74 ± 0.25	4.58 ± 0.64	4.69 ± 0.82	4.00 ± 0.46	−0.56*	−0.52*	−0.75*	0.042
	26.48	Mannose	6.44 ± 0.46	5.37 ± 0.43	4.12 ± 0.66	4.07 ± 0.64	−0.26	−0.64*	−0.66*	0.042
	29.21	Tagatose	6.79 ± 1.06	5.18 ± 0.57	5.38 ± 1.31	2.64 ± 0.43	−0.39	−0.34	−1.36*	0.045
	24.53	Xylose	4.96 ± 0.35	4.15 ± 0.47	4.03 ± 0.58	6.86 ± 0.81	−0.26	−0.3	0.47*	0.017
**Others**	15.04	1-(ethenyloxy)-3-methylbenzene	3.59 ± 0.66	6.20 ± 0.67	6.56 ± 0.47	3.65 ± 0.79	0.79*	0.87*	0.02	0.018
	24.14	1,3,5-Benzetriol	13.44 ± 5.19	3.37 ± 0.48	2.37 ± 0.30	0.82 ± 0.17	−2.00*	−2.50*	−4.03*	4.86E-06
	40.16	5-Methyluridine	4.14 ± 0.48	4.38 ± 0.66	3.55 ± 0.35	7.92 ± 0.85	0.08	−0.22	0.94*	0.003
	47.08	5-O-C-D-quinic acid	7.24 ± 1.70	6.30 ± 0.79	3.48 ± 0.38	2.98 ± 0.30	−0.2	−1.06*	−1.28*	0.005
	15.69	Cadaverine	2.49 ± 0.13	5.55 ± 1.23	5.54 ± 0.62	6.43 ± 0.71	1.16*	1.16*	1.37*	0.004
	10.57	Cerulenin	4.15 ± 0.31	4.75 ± 0.67	4.87 ± 0.34	6.24 ± 0.32	0.20	0.23	0.59*	0.032
	28.33	Epicatechin	11.67 ± 2.14	2.24 ± 0.89	3.16 ± 1.01	2.93 ± 1.15	−2.38*	−1.89*	−1.99*	0.011
	20.46	Ethanolamine	3.24 ± 0.5	4.45 ± 0.71	5.13 ± 0.89	7.18 ± 0.67	0.46	0.67	1.15*	0.015
	36.04	Methyl galactoside	4.78 ± 0.68	4.24 ± 0.59	3.35 ± 0.15	7.64 ± 0.44	−0.17	−0.51*	0.68*	4.30E-04
	32.34	Oleamide	8.98 ± 1.96	4.85 ± 1.96	1.58 ± 0.17	4.59 ± 1.15	−0.89	−2.50*	−0.97	0.002
	21.56	Putrescine	5.52 ± 0.83	5.38 ± 1.26	2.64 ± 0.53	6.46 ± 1.20	−0.04	−1.07	0.23	0.041

*Values are means of five replicates ±SE. Fold changes are calculated using the formula log2 (Control/Treatment). *indicates significance (*p* < 0.05); **indicates high significance (*p* < 0.01).

**Table 2:** Relative concentrations and fold changes of significant metabolites in the leaves of *B*.*brachycarpa* under different levels of watering treatments.

Metabolite groups	RT	Metabolites	Relative concentrations				Fold changes			*p*
			CK	T1	T2	T3	FC^T1/CK^	FC^T2/CK^	FC^T3/CK^	
**Amino acids**	10.20	Alanine	9.15 ± 2.37	3.80 ± 0.97	3.91 ± 1.02	3.14 ± 0.35	−2.16*	−2.09*	−2.64*	0.043
	21.23	Aspartic acid	10.47 ± 0.58	2.80 ± 0.89	3.46 ± 0.89	3.27 ± 0.93	−3.25*	−2.73*	−2.87*	0.028
	13.93	Beta-Alanine	7.23 ± 1.17	6.84 ± 2.06	2.76 ± 0.37	3.17 ± 0.62	−0.14	−2.38*	−2.04*	0.019
	07.30	Dimethylglycine	12.24 ± 0.35	2.48 ± 1.65	4.40 ± 0.41	0.88 ± 1.42	−3.94*	−2.52*	−6.49*	5.77E-05
	21.43	GABA	4.94 ± 2.86	10.63 ± 0.66	2.17 ± 1.20	2.26 ± 1.27	1.89*	−2.03	−1.93	0.003
	23.59	Glutamic acid	7.88 ± 3.85	3.87 ± 0.50	4.24 ± 1.70	4.01 ± 0.07	−1.75*	−1.53*	−1.67*	0.049
	15.37	Isoleucine	3.96 ± 1.36	9.77 ± 1.27	3.91 ± 0.64	2.37 ± 0.9	2.23*	−0.03	−1.26	0.013
	23.66	Phenylalanine	8.51 ± 1.33	3.84 ± 0.80	4.14 ± 1.03	3.51 ± 1.03	−1.96*	−1.78*	−2.18*	0.021
	14.47	Serine	7.09 ± 0.82	4.51 ± 0.56	3.26 ± 0.66	5.14 ± 0.68	−1.12*	−1.91*	−0.79*	0.011
	17.84	Threonine	8.62 ± 0.46	5.26 ± 1.30	2.36 ± 0.34	3.75 ± 1.13	−1.22*	−3.19**	−2.06*	0.003
	13.27	Valine	8.58 ± 2.61	4.91 ± 1.20	3.96 ± 0.94	2.55 ± 0.57	−1.37	−1.9*	−2.99*	0.049
**Organic acids**	30.41	4-Coumaric acid	3.36 ± 0.88	6.16 ± 0.72	2.85 ± 0.48	7.62 ± 0.49	1.49*	−0.4	2.02*	0.015
	11.04	Acrylic acid	7.30 ± 0.53	5.42 ± 1.16	4.00 ± 0.79	3.28 ± 0.54	−0.73*	−1.48**	−1.97**	0.004
	30.49	Ascorbic acid	8.48 ± 0.48	3.47 ± 1.39	4.37 ± 0.96	3.68 ± 0.89	−2.20*	−1.63*	−2.06*	0.018
	19.92	Butyric acid	8.89 ± 0.46	4.80 ± 1.24	2.93 ± 1.10	3.38 ± 1.33	−1.52*	−2.73*	−2.38*	0.027
	34.11	Caffeic acid	2.42 ± 0.36	5.25 ± 1.70	4.52 ± 1.20	7.81 ± 1.10	1.9	1.54	2.88*	0.033
	27.95	Citric acid	3.05 ± 0.80	8.53 ± 1.11	4.36 ± 0.82	4.07 ± 0.38	2.54*	0.88	0.71	0.046
	38.20	Galacturonic acid	8.52 ± 1.58	4.07 ± 1.16	4.09 ± 0.54	3.32 ± 0.94	−1.82*	−1.81*	−2.32*	0.016
	9.50	Glycolic acid	7.17 ± 0.61	4.72 ± 0.93	4.35 ± 0.55	3.76 ± 0.70	−1.03*	−1.23*	−1.59**	0.033
	9.05	Lactic Acid	3.87 ± 0.46	4.27 ± 1.32	8.57 ± 1.58	3.29 ± 0.70	0.24	1.96*	−0.41	0.026
	53.87	Lignoceric acid	10.48 ± 1.45	2.08 ± 0.69	4.09 ± 0.84	3.35 ± 0.61	−3.98*	−2.32*	−2.81*	4.82E-04
	35.42	Linoleic acid	4.41 ± 0.57	7.53 ± 0.86	4.48 ± 0.68	3.58 ± 0.60	1.32*	0.04	−0.52	0.011
	12.98	Malonic acid	8.70 ± 1.16	6.29 ± 1.56	2.65 ± 0.43	2.36 ± 0.43	−0.80	−2.93*	−3.22*	0.001
	18.11	Mesoxalic acid	7.23 ± 0.73	7.83 ± 2.04	2.28 ± 0.51	2.66 ± 0.24	0.20	−2.85*	−2.47*	8.53E-05
	32.47	Palmitic Acid	3.29 ± 0.29	5.29 ± 1.02	6.87 ± 0.87	4.55 ± 0.57	1.17*	1.82**	0.80*	0.013
	9.79	Pyruvic acid	7.45 ± 0.98	4.78 ± 1.43	4.63 ± 0.51	3.14 ± 0.43	−1.10*	−1.17*	−2.13*	0.039
	15.99	Succinic acid	6.41 ± 0.48	5.81 ± 1.05	4.42 ± 0.52	3.36 ± 0.61	−0.24	−0.92*	−1.59*	0.048
	22.18	Threonic acid	7.26 ± 1.05	4.62 ± 0.83	4.64 ± 0.79	3.49 ± 0.71	−1.11*	−1.10*	−1.81*	0.046
	35.54	α-Linolenic acid	3.94 ± 1.17	7.27 ± 0.91	4.54 ± 1.02	4.25 ± 0.53	1.51*	0.35	0.18	0.044
**Sugar alcohols**	26.23	Mannitol	4.69 ± 0.47	6.48 ± 0.22	5.20 ± 0.66	3.63 ± 0.73	0.80*	0.25	−0.64	0.049
	20.75	Threitol	7.76 ± 0.63	3.95 ± 1.12	4.98 ± 0.97	3.31 ± 0.63	−1.67*	−1.09*	−2.10*	0.047
**Sugars**	34.04	Allose	7.37 ± 0.23	3.75 ± 0.50	4.82 ± 0.83	4.06 ± 0.72	−1.67*	−1.05*	−1.47*	0.019
	24.53	Arabinose	6.73 ± 0.41	5.39 ± 0.89	3.90±0.50	3.98 ± 0.56	−0.55*	−1.34**	−1.29**	0.036
	20.24	Erythrulose	7.40 ± 0.70	4.30 ± 0.63	4.01 ± 0.49	4.29 ± 0.59	−1.34*	−1.51*	−1.34*	0.013
	29.23	Fructose	7.61 ± 1.21	4.59 ± 0.84	3.05 ± 0.73	4.74 ± 1.12	−1.25*	−2.25**	−1.17*	0.043
	29.38	Galactose	7.22 ± 0.76	4.93 ± 0.80	4.03 ± 0.76	3.82 ± 0.78	−0.94*	−1.44*	−1.57*	0.047
	38.38	Glucose	8.81 ± 1.12	4.40 ± 0.95	3.10 ± 0.73	3.69 ± 0.94	−1.71*	−2.58*	−2.14*	0.013
	25.06	Lyxose	8.19 ± 1.06	4.65 ± 0.79	3.32 ± 0.50	3.84 ± 1.03	−1.39*	−2.23*	−1.87*	0.014
	46.43	Maltose	1.80 ± 0.62	6.92 ± 1.64	3.56 ± 1.19	7.72 ± 2.51	3.31	1.68	3.59*	0.043
	32.09	Rhamnose	8.16 ± 1.52	3.79 ± 0.74	4.55 ± 0.85	3.50 ± 0.21	−1.89*	−1.44*	−2.09*	0.025
	41.28	Turanose	8.55 ± 2.33	4.98 ± 1.05	4.13 ± 0.53	2.35 ± 0.83	−1.33*	−1.80*	−3.19*	0.013
	24.38	Xylose	7.06 ± 0.56	4.18 ± 0.64	4.73 ± 0.61	4.02 ± 0.65	−1.29*	−0.99*	−1.39*	0.034
**Others**	45.81	Catechine	10.72 ± 1.99	2.01 ± 0.94	4.11 ± 1.04	3.17 ± 1.03	−2.42*	−1.38*	−1.76*	0.021
	10.59	Cerulenin	7.04 ± 0.66	5.24 ± 0.98	4.01 ± 0.56	3.71 ± 0.35	−0.43*	−0.81*	−0.93**	0.025
	14.65	Ethanolamine	4.44 ± 0.80	7.50 ± 0.71	4.33 ± 0.61	3.73 ± 0.59	1.29*	−0.07	−0.43	0.016
	34.72	Methyl galactoside	8.26 ± 1.11	3.86 ± 0.65	3.60 ± 0.89	4.28 ± 0.67	−1.10*	−1.20*	−0.95*	0.016
	38.83	Oleamide	8.46 ± 0.78	3.78 ± 1.04	4.03 ± 0.95	3.73 ± 0.60	−1.99*	−1.83*	−2.02*	0.045
	21.82	α-Bisabolene	2.29 ± 0.69	7.79 ± 0.90	3.37 ± 0.49	6.55 ± 1.68	1.77*	0.55	1.51*	0.041
	49.09	α-Tocopherol	7.80 ± 1.17	2.85 ± 0.51	4.76 ± 1.16	4.59 ± 0.72	−2.48*	−1.21*	−1.31*	0.023
	21.05	β-Bisabolene	2.23 ± 0.72	7.45 ± 0.92	3.56 ± 0.52	6.76 ± 1.72	1.74*	0.67	1.60*	0.043

*Values are means of five replicates ±SE. Fold changes are calculated using the formula log2 (Control/Treatment). *indicates significance (*p* < 0.05); **indicates high significance (*p* < 0.01).

**Table 3:** Relative concentrations and fold changes of significant metabolites in the leaves of *C*. *distans* under different levels of watering treatments.

Metabolite groups	RT	Metabolites	Relative concentrations				Fold changes			*p*
			CK	T1	T2	T3	FC^T1/CK^	FC^T2/CK^	FC^T3/CK^	
**Amino acids**	21.44	GABA	3.35 ± 0.50	4.61 ± 0.62	4.56 ± 1.09	7.48 ± 0.54	0.46	0.44	1.16*	0.027
	15.38	Isoleucine	3.52 ± 0.51	4.57 ± 0.83	8.00 ± 0.55	3.91 ± 1.03	0.38	1.18*	0.15	0.039
	11.66	Leucine	4.71 ± 0.86	7.34 ± 0.66	3.33 ± 0.78	4.62 ± 1.00	0.64*	−0.5	−0.03	0.048
	30.08	Lysine	3.20 ± 0.60	8.24 ± 1.54	4.78 ± 0.99	3.78 ± 0.59	1.36*	0.58	0.24	0.025
	21.16	Methionine	4.29 ± 0.78	8.21 ± 1.30	4.31 ± 0.88	3.18 ± 0.53	0.94*	0.01	−0.43	0.033
	23.67	Phenylalanine	3.64 ± 0.52	8.04 ± 1.55	4.29 ± 0.93	4.03 ± 0.21	1.14*	0.24	0.15	0.041
	14.45	Serine	3.89 ± 0.65	7.68 ± 1.38	5.28 ± 1.13	3.15 ± 0.84	0.98*	0.44	−0.3	0.027
	30.38	Tyrosine	3.63 ± 0.56	5.60 ± 0.49	6.18 ± 0.41	4.58 ± 0.39	0.63*	0.77**	0.34	0.006
**Organic acids**	19.32	2-Pentenedioic acid	1.92 ± 0.44	7.81 ± 2.27	3.36 ± 0.51	6.91 ± 0.42	2.02**	0.81	1.85*	0.001
	32.92	3-Heptenedioic acid	3.09 ± 0.42	8.09 ± 1.30	5.21 ± 1.00	3.61 ± 0.68	1.39*	0.75	0.22	0.028
	27.31	4-Coumaric acid	1.95 ± 0.20	7.59 ± 1.22	4.63 ± 1.48	5.84 ± 2.39	1.96*	1.25	1.58	0.012
	27.58	Galactaric acid	2.96 ± 0.40	7.35 ± 1.22	6.78 ± 1.82	2.91 ± 0.32	1.31*	1.20*	−0.02	0.006
	16.38	Glyceric acid	3.19 ± 0.50	8.34±1.82	3.72 ± 1.00	4.75 ± 0.39	1.39*	0.22	0.57	0.014
	9.46	Glycolic acid	2.96 ± 0.45	7.56 ± 2.09	3.74 ± 0.54	5.73 ± 0.36	1.35*	0.34	0.95	0.017
	16.72	Itaconic acid	2.08 ± 0.41	6.41 ± 2.66	4.59 ± 1.34	6.92 ± 0.71	1.62	1.14	1.73*	0.048
	45.21	Lignoceric acid	1.81 ± 0.36	7.45 ± 2.91	3.60 ± 0.78	7.14 ± 1.16	2.04	0.99	1.98	0.009
	35.45	Linoleic acid	2.17 ± 0.24	5.60 ± 1.18	6.23 ± 1.62	6.01 ± 0.62	1.37	1.52*	1.47*	0.013
	16.87	Methylmaleic acid	2.86 ± 0.37	4.53 ± 0.84	4.92 ± 1.09	7.69 ± 0.35	0.66	0.78	1.43*	0.013
	35.97	N-Acetylneuraminic acid	3.54 ± 0.48	6.61 ± 0.18	4.61 ± 0.79	5.23 ± 0.67	0.90	0.38*	0.56	0.029
	32.49	Palmitic Acid	2.14 ± 0.22	5.14 ± 1.33	6.46 ± 1.43	6.26 ± 0.81	1.26	1.59*	1.55*	0.008
	28.72	Quinic acid	7.20 ± 0.71	5.33 ± 0.96	4.35 ± 1.43	3.12 ± 0.15	−0.43	−0.73*	−1.21*	0.044
	31.33	Sebacic acid	3.55 ± 0.35	8.30 ± 1.77	3.94 ± 0.54	4.21 ± 0.72	1.23*	0.15	0.25	0.014
	27.81	Shikimic acid	3.72 ± 0.62	7.01 ± 0.99	4.00 ± 0.65	5.28 ± 0.44	0.91*	0.10	0.51	0.025
	36.04	Stearic acid	2.83 ± 0.42	4.77 ± 0.68	7.18 ± 0.44	5.22 ± 0.59	0.75*	1.34**	0.88*	5.18E-04
	35.56	α-Linolenic acid	2.38 ± 0.48	5.88 ± 1.43	5.01 ± 0.97	6.73 ± 1.02	1.30	1.07	1.50*	0.017
**Sugar alcohols**	25.49	Arabitol	2.33 ± 0.37	4.25 ± 0.59	6.52 ± 0.90	6.90 ± 0.63	0.87*	1.48**	1.57**	1.03E-04
	38.73	Galactinol	2.28 ± 0.28	8.52 ± 1.60	4.46 ± 1.31	4.75 ± 0.58	1.90*	0.97	1.06	0.007
	29.09	Mannitol	4.83 ± 1.46	8.22 ± 1.28	3.52 ± 0.36	3.44 ± 0.62	0.77*	−0.46	−0.49	0.036
	21.48	Ribitol	3.02 ± 0.20	5.14 ± 1.06	6.14 ± 0.90	5.70 ± 0.53	0.77	1.02*	0.92*	0.032
	20.93	Threitol	2.28 ± 0.17	3.40 ± 0.66	7.57 ± 2.19	6.75 ± 0.66	0.58	1.73**	1.57*	9.18E-04
**Sugars**	30.28	Fructose	3.71 ± 0.42	6.78 ± 1.00	4.94 ± 0.68	4.57 ± 0.31	0.87*	0.41	0.3	0.036
	29.85	Galactose	3.01 ± 0.21	8.13 ± 2.20	5.08 ± 1.04	3.78 ± 0.31	1.43*	0.76	0.33	0.019
	31.88	Glucose 6-O-à-D-GP	2.77 ± 0.30	7.91 ± 2.11	4.06 ± 0.49	5.25 ± 0.78	1.51	0.55	0.92	0.01
	25.41	Levoglucosan	2.90 ± 0.39	4.74 ± 0.51	8.06 ± 1.74	4.30 ± 0.59	0.71	1.47*	0.57	0.005
	23.73	Rhamnose	2.09 ± 0.18	5.73 ± 1.58	3.48 ± 0.60	8.71 ± 1.06	1.46*	0.74	2.06**	4.22E-04
	49.01	Melibiose	3.11 ± 0.27	7.52 ± 1.80	4.52 ± 0.56	4.85 ± 0.38	1.27*	0.54	0.64	0.015
	26.86	Tagatofuranose	2.03 ± 0.45	3.53 ± 0.67	8.12 ± 1.43	6.32 ± 0.84	0.8	2.00**	1.64*	2.31E-04
	31.42	Turanose	2.62 ± 0.49	7.72 ± 1.38	5.36 ± 1.08	4.29 ± 0.69	1.56*	1.03	0.71	0.014
	33.71	α-D-Glucopyranose	3.78 ± 0.12	7.02 ± 0.92	5.38 ± 1.08	3.82 ± 0.28	0.89*	0.51	0.02	0.02
	23.34	β-D-Xylopyranose	2.64 ± 0.22	4.83 ± 0.81	6.21 ± 0.89	6.32 ± 0.68	0.87*	1.23*	1.26*	0.002
	45.51	β-Gentiobiose	3.51 ± 0.55	5.08 ± 0.69	6.48 ± 0.91	4.93 ± 0.61	0.53*	0.88**	0.49*	0.042
**Others**	42.00	2-Deoxyadenosine	3.22 ± 0.44	9.39 ± 2.61	3.21 ± 0.55	4.18 ± 0.74	1.54*	0	0.38	0.017
	13.64	Borneol	0.54 ± 0.24	3.59 ± 1.09	8.81 ± 2.46	7.06 ± 1.97	2.73	4.03**	3.71*	8.92E-04
	50.59	Campesterol	3.14 ± 0.31	8.04 ± 1.46	4.14 ± 0.76	4.69 ± 0.18	1.36*	0.4	0.58	0.005
	41.29	Inosine	2.87 ± 0.41	7.28 ± 0.72	4.54 ± 1.05	5.31 ± 0.97	1.34**	0.66	0.89*	0.017
	13.79	Linalool	2.98 ± 1.14	2.50 ± 0.87	4.60 ± 1.45	9.93 ± 2.08	−0.25	0.63	1.74*	0.048
	49.28	Magnolol	3.27 ± 0.44	9.08 ± 3.48	2.98 ± 0.47	4.68 ± 0.73	1.47*	−0.13	0.52	0.014
	22.41	Methyl acetopyruvate	2.46 ± 0.61	5.30 ± 1.37	4.78 ± 1.54	7.46 ± 1.18	1.11	0.96	1.60*	0.044
	18.30	Methyleugenol	2.49 ± 0.55	4.51 ± 1.29	6.49 ± 1.32	6.52 ± 0.86	0.86	1.38*	1.39*	0.037
	33.00	N-Acetyl-D-glucosamine	3.25 ± 0.57	5.36 ± 0.55	6.80 ± 0.62	4.59 ± 0.37	0.72*	1.07**	0.5	0.003
	38.85	Oleamide	3.86 ± 0.44	6.41 ± 0.22	4.47 ± 0.67	5.26 ± 0.57	0.73*	0.21	0.45	0.037
	50.98	Stigmasterol	3.29 ± 0.30	8.27 ± 1.75	4.59 ± 0.98	3.85 ± 0.47	1.33*	0.48	0.23	0.03
	16.24	α-Terpineol	1.87 ± 0.55	4.42 ± 1.53	5.26 ± 1.52	8.45 ± 1.70	1.24	1.49	2.18*	0.012
	51.96	β-Sitosterol	3.61 ± 0.27	7.70 ± 1.77	4.54 ± 0.63	4.16 ± 0.16	1.09*	0.33	0.2	0.031

*Values are means of five replicates ±SE. Fold changes are calculated using the formula log2 (Control/Treatment). *indicates significance (*p* < 0.05); **indicates high significance (*p* < 0.01).

**Table 4:** Relative concentrations and fold changes of significant metabolites in the leaves of *A*. *sitosa* under different levels of watering treatments.

Metabolite groups	RT	Metabolites	Relative concentrations				Fold changes			*p*
			CK	T1	T2	T3	FC^T1/CK^	FC^T2/CK^	FC^T3/CK^	
**Amino acids**	21.18	5-Oxoproline	5.55 ± 0.45	8.29 ± 1.94	3.01 ± 0.74	3.16 ± 0.36	0.58	−0.88*	−0.81*	0.005
	10.17	Alanine	6.86 ± 0.35	9.17 ± 3.66	1.79 ± 0.82	2.19 ± 0.61	0.42	−1.94	−1.65	0.036
	21.23	Aspartic acid	4.68 ± 0.95	8.74 ± 1.99	4.41 ± 1.88	2.17 ± 1.00	0.9	−0.09	−1.11	0.024
	19.65	Cystathionine	6.28 ± 0.88	8.90 ± 3.03	2.38 ± 0.46	2.43 ± 0.59	0.5	−1.40*	−1.37*	0.023
	23.60	Dimethylglycine	5.25 ± 1.69	7.96 ± 1.90	2.33 ± 0.57	4.45 ± 0.86	0.6	−1.17	−0.24	0.019
	11.69	Glutamic acid	4.65 ± 0.66	9.73 ± 3.21	3.26 ± 1.21	2.36 ± 0.68	1.07*	−0.51	−0.98	0.022
	30.04	Leucine	7.74 ± 1.59	8.86 ± 3.45	1.45 ± 0.24	1.95 ± 0.66	0.19	−2.42*	−1.99*	0.018
	21.15	Lysine	5.30 ± 0.90	9.96 ± 3.63	2.44 ± 0.79	2.30 ± 0.59	0.91	−1.12	−1.2	0.005
	7.23	Methionine	6.88 ± 1.48	7.17 ± 1.26	2.86 ± 0.37	3.09 ± 1.14	0.06	−1.27*	−1.15*	0.039
	23.66	Phenylalanine	6.66 ± 1.20	8.26 ± 2.37	2.56 ± 0.49	2.52 ± 0.58	0.31	−1.38*	−1.40*	0.013
	42.80	Tyrosine	5.70 ± 0.96	9.21 ± 3.18	2.50 ± 0.38	2.60 ± 0.42	0.69	−1.19	−1.13	0.004
**Organic acids**	28.43	2-Ketoglutaric acid	6.48 ± 0.59	8.19 ± 1.91	2.34 ± 0.25	2.99 ± 0.23	0.34	−1.47*	−1.12*	1.31E-04
	9.53	2-Methyl-4-pentenoic acid	6.14 ± 0.67	7.69 ± 3.10	1.93 ± 0.41	4.23 ± 0.97	0.32	−1.67	−0.54	0.017
	31.25	2-Sebacic acid	5.81 ± 1.90	10.23 ± 5.1	1.52 ± 0.22	2.44 ± 0.21	0.82	−1.93	−1.25	0.003
	32.03	3-Heptenedioic acid	6.39 ± 1.62	9.95 ± 3.60	1.42 ± 0.26	2.24 ± 0.12	0.64	−2.17	−1.51	0.001
	44.82	3-Hydroxymandelic acid	6.17 ± 0.86	7.28 ± 2.02	2.53 ± 0.32	4.02 ± 0.88	0.24	−1.29	−0.62	0.025
	30.39	4-Coumaric acid	6.28 ± 1.56	7.75 ± 2.67	2.40 ± 0.47	3.57 ± 0.77	0.3	−1.39	−0.81	0.05
	22.41	Acetopyruvic acid	5.16 ± 1.91	9.64 ± 2.01	2.51 ± 0.43	2.69 ± 0.83	0.9	−1.04	−0.94	0.009
	26.55	Aconitic acid (E)	5.60 ± 1.25	8.93 ± 2.37	3.11 ± 0.80	2.36 ± 0.36	0.67	−0.85	−1.25	0.008
	6.91	Acrylic acid	6.23 ± 0.87	7.83 ± 2.45	2.55 ± 0.40	3.39 ± 0.38	0.33	−1.29	−0.88	0.017
	30.48	Ascorbic acid	4.89 ± 1.40	9.32 ± 2.69	4.10 ± 1.23	1.68 ± 0.42	0.93	−0.25	−1.54	0.008
	34.11	Caffeic acid	5.22 ± 0.51	8.33 ± 3.09	2.36 ± 0.55	4.09 ± 0.64	0.67	−1.15	−0.35	0.043
	24.39	Dodecanoic acid	5.42 ± 0.76	8.83 ± 2.71	2.50 ± 0.26	3.25 ± 0.23	0.7	−1.12	−0.74	7.62E-04
	16.97	Fumaric acid	6.61 ± 1.49	8.05 ± 1.73	2.17 ± 0.28	3.17 ± 0.35	0.28	−1.61*	−1.06*	3.71E-04
	27.58	Galactaric acid	4.74 ± 0.66	9.11 ± 2.87	2.30 ± 0.25	3.84 ± 0.42	0.94	−1.04	−0.3	0.002
	31.75	Gluconic acid	4.62 ± 0.76	9.54 ± 3.07	2.87 ± 0.43	2.98 ± 0.24	1.05*	−0.69	−0.63	0.018
	16.38	Glyceric acid	4.74 ± 1.37	9.87 ± 1.63	2.18 ± 0.42	3.21 ± 0.80	1.06*	−1.12	−0.56	7.37E-04
	9.47	Glycolic acid	5.61 ± 1.24	7.82 ± 1.96	2.33 ± 0.38	4.24 ± 0.60	0.48	−1.27	−0.4	0.005
	33.28	Isoferulic acid	6.37 ± 1.48	8.39 ± 2.17	2.32 ± 0.46	2.92 ± 0.41	0.4	−1.46	−1.13	0.009
	16.71	Itaconic acid	4.09 ± 0.98	8.10 ± 1.67	2.72 ± 0.49	5.09 ± 1.64	0.99	−0.59	0.32	0.032
	45.20	Lignoceric acid	6.10 ± 0.71	8.71 ± 3.50	2.18 ± 0.25	3.01 ± 0.41	0.51	−1.48	−1.02	0.014
	20.49	Malic acid	6.18 ± 2.59	9.13 ± 2.68	2.52 ± 0.71	2.17 ± 0.37	0.56	−1.29	−1.51	0.042
	15.42	Niacin	6.68 ± 1.08	7.17 ± 1.76	2.67 ± 0.30	3.48 ± 0.45	0.1	−1.32*	−0.94*	0.002
	11.21	Oxalic acid	5.58 ± 0.70	9.46 ± 2.91	1.73 ± 0.30	3.24 ± 0.48	0.76	−1.69	−0.78	4.65E-04
	8.76	Propanoic acid	4.29 ± 0.88	8.53 ± 1.71	2.36 ± 0.27	4.82 ± 1.16	0.99*	−0.86	0.17	0.007
	28.71	Quinic acid	7.53 ± 0.69	7.91 ± 1.71	1.87 ± 0.49	2.69 ± 0.55	0.07	−2.01*	−1.49*	2.06E-04
	27.80	Shikimic acid	7.35 ± 0.95	7.46 ± 2.49	2.09 ± 0.45	3.10 ± 0.43	0.02	−1.81*	−1.25*	0.003
	24.84	Stearic acid	5.07 ± 0.75	8.76 ± 1.94	2.67 ± 0.56	3.50 ± 0.47	0.79*	−0.93	−0.53	0.004
	15.98	Succinic acid	5.55 ± 0.99	8.29 ± 2.37	2.33 ± 0.38	3.82 ± 0.52	0.58	−1.25	−0.54	0.003
	21.76	Threonic acid	5.54 ± 0.76	8.54 ± 2.22	2.55 ± 0.19	3.37 ± 0.33	0.62	−1.12	−0.72	0.002
	41.60	Glucopyranuronic acid	11.25 ± ± 2.73	4.18 ± 1.30	2.19 ± 0.51	2.38 ± 0.66	−1.43*	−2.36*	−2.24*	0.005
**Sugar alcohols**	25.92	Arabitol	3.67 ± 0.98	3.13 ± 2.85	6.27 ± 0.45	6.94 ± 0.54	−0.23	0.77*	0.92*	6.68E-04
	6.96	Ethylene glycol	6.20 ± 1.95	7.78 ± 1.27	2.73 ± 3.51	3.29 ± 0.71	0.33	−1.18	−0.91	0.003
	30.35	Dulcitol	6.59 ± 1.35	8.57 ± 1.22	2.27 ± 0.35	2.57 ± 0.37	0.38	−1.54	−1.36	0.005
	47.08	Galactinol	6.60 ± 2.67	7.68 ± 1.22	2.31 ± 0.83	3.41 ± 2.28	0.22	−1.51*	−0.95*	2.21E-04
	14.89	Glycerol	5.27 ± 0.39	8.41 ± 0.63	3.17 ± 0.60	3.15 ± 0.37	0.67	−0.73	−0.74	0.033
	33.18	Myo-Inositol	5.57 ± 0.41	7.52 ± 2.32	3.15 ± 0.33	3.76 ± 0.20	0.43	−0.82	−0.57	0.02
**Sugars**	40.86	3-α-Mannobiose	6.33 ± 0.84	7.60 ± 2.08	3.01 ± 0.78	3.05 ± 0.55	0.26	−1.07	−1.05	0.021
	33.80	Altrose	6.77 ± 0.94	7.02 ± 1.56	2.74 ± 0.46	3.47 ± 0.67	0.05	−1.30*	−0.96	0.012
	24.53	Arabinose	5.10 ± 0.59	9.13 ± 3.26	2.51 ± 0.31	3.26 ± 0.31	0.84	−1.02	−0.65	0.002
	35.15	Cellobiose	5.96 ± 0.70	8.08 ± 2.45	2.17 ± 0.37	3.80 ± 0.63	0.44	−1.46**	−0.65*	0.006
	37.18	Floridoside	6.56 ± 1.63	7.47 ± 2.46	2.41 ± 0.43	3.57 ± 0.40	0.19	−1.44	−0.88	0.022
	29.39	Galactose	5.78 ± 0.72	7.49 ± 1.49	3.06 ± 0.47	3.68 ± 0.81	0.37	−0.92	−0.65	0.011
	32.09	Glucose	6.64 ± 0.92	7.90 ± 1.48	2.57 ± 0.22	2.88 ± 0.96	0.25	−1.37*	−1.21*	0.013
	31.84	Glucose, 6-O-à-D-GP	5.80 ± 0.71	9.15 ± 1.88	1.78 ± 0.50	3.27 ± 0.65	0.66	−1.7	−0.83	0.008
	40.24	Maltose	6.64 ± 0.72	7.93 ± 2.23	2.23 ± 0.29	3.21 ± 0.45	0.26	−1.57**	−1.05*	0.002
**Sugars**	48.99	Melibiose	7.65 ± 2.19	7.84 ± 2.66	1.75 ± 0.26	2.76 ± 0.21	0.04	−2.13*	−1.47	0.001
	33.94	Sedoheptulose	6.39 ± 1.17	7.69 ± 1.45	2.78 ± 0.38	3.14 ± 0.51	0.27	−1.20**	−1.03*	0.003
	23.33	Talose	6.12 ± 0.54	7.57 ± 1.88	3.24 ± 0.39	3.07 ± 0.31	0.31	−0.92	−1	0.002
	20.24	Threose	7.36 ± 0.93	8.33 ± 2.14	1.71 ± 0.22	2.61 ± 0.24	0.18	−2.11*	−1.50*	3.46E-05
	43.57	Trehalose	7.61 ± 2.07	7.89 ± 2.35	2.35 ± 0.47	2.16 ± 0.31	0.05	−1.70*	−1.82*	0.01
	36.03	Turanose	7.84 ± 1.74	7.94 ± 2.03	2.36 ± 0.51	1.86 ± 0.52	0.02	−1.73*	−2.08*	0.004
	27.12	Xylofuranose	6.33 ± 0.87	7.36 ± 1.29	2.72 ± 0.14	3.59 ± 0.65	0.22	−1.22*	−0.82*	0.001
	26.74	Xylose	5.43 ± 1.00	8.30 ± 2.35	2.70 ± 0.70	3.57 ± 0.39	0.61	−1.01	−0.61	0.004
	27.00	α-D-fructofuranoside	5.15 ± 0.67	9.68 ± 3.24	2.38 ± 0.49	2.79 ± 0.33	0.91	−1.11	−0.88	0.004
	31.15	β-D-Glucopyranose	4.87 ± 0.44	8.34 ± 2.04	2.79 ± 0.32	4.00 ± 0.19	0.78	−0.8	−0.28	0.005
	42.75	β-Gentiobiose	6.12 ± 0.83	9.64 ± 4.92	2.13 ± 0.65	2.10 ± 0.72	0.66	−1.52	−1.54	0.045
**Others**	49.21	1-Octacosanol	10.7 ± 2.29	5.20 ± 2.36	2.56 ± 0.48	1.54 ± 0.29	−1.04*	−2.06*	−2.80*	9.34E-04
	43.48	2-linoleoylglycerol	6.58 ± 1.03	8.29 ± 2.82	2.57 ± 0.52	2.56 ± 0.32	0.33	−1.36	−1.36	0.005
	46.98	4-Hydroxy-3-methoxyphenylglycol	7.99 ± 1.67	8.56 ± 5.20	1.75 ± 0.37	1.69 ± 0.58	0.1	−2.19	−2.24	0.012
	38.49	5-Methyluridine	6.58 ± 1.26	8.72 ± 2.05	1.87 ± 0.18	2.84 ± 0.29	0.41	−1.82*	−1.21*	4.18E-05
	49.08	Alpha-Tocopherol	9.43 ± 0.62	4.58 ± 1.99	2.02 ± 0.92	3.96 ± 0.91	−1.04	−2.22*	−1.25	0.048
	41.47	Arbutin	5.96 ± 0.82	8.81 ± 3.10	2.23 ± 0.44	3.00 ± 0.63	0.56	−1.42	−0.99	0.021
	50.58	Campesterol	5.81 ± 0.58	8.86 ± 3.12	2.25 ± 0.31	3.08 ± 0.18	0.61	−1.37	−0.92	0.001
	26.72	Glycerol 3-phosphate	6.47 ± 2.00	8.36 ± 3.31	2.87 ± 0.31	2.30 ± 0.48	0.37	−1.17	−1.49	0.007
	44.22	Guanosine	8.30 ± 1.90	7.07 ± 1.82	2.05 ± 0.33	2.58 ± 0.69	−0.23	−2.02*	−1.69*	0.002
	25.22	Methyl galactoside	5.95 ± 0.92	8.09 ± 3.45	2.44 ± 0.33	3.51 ± 0.66	0.44	−1.29	−0.76	0.015
	25.39	N-Acetyl glucosamine	5.30 ± 0.92	10.26 ± 2.7	2.00 ± 0.58	2.44 ± 0.55	0.95	−1.41	−1.12	0.032
	35.96	Oleamide	6.58 ± 0.95	9.22 ± 2.39	1.51 ± 0.31	2.69 ± 0.25	0.49	−2.12*	−1.29*	3.78E-05
	14.79	Phosphate	4.43 ± 0.74	8.35 ± 2.87	2.96 ± 0.38	4.26 ± 0.49	0.91	−0.58	−0.06	0.028
	12.21	Phosphoric acid monomethyl ester	3.91 ± 0.78	9.46 ± 2.35	3.07 ± 0.42	3.56 ± 0.60	1.27*	−0.35	−0.14	0.009
	34.79	Phytol	6.06 ± 1.19	8.00 ± 2.31	1.98 ± 0.19	3.96 ± 0.38	0.4	−1.61	−0.61	0.001
	52.14	Stigmastanol	7.84 ± 1.71	7.98 ± 2.23	1.75 ± 0.31	2.42 ± 0.18	0.03	−2.16*	−1.70*	6.66E-04
	50.96	Stigmasterol	5.85 ± 0.87	8.89 ± 2.70	2.29 ± 0.29	2.96 ± 0.17	0.6	−1.35	−0.98	0.002
	14.07	Urea	4.75 ± 0.52	6.89 ± 2.35	6.59 ± 0.22	1.77 ± 0.40	0.54	0.47	−1.42	0.049
	51.93	β-Sitosterol	6.48 ± 1.07	8.57 ± 2.99	2.11 ± 0.27	2.84 ± 0.21	0.4	−1.62	−1.19	7.08E-04

*Values are means of five replicates ±SE. Fold changes are calculated using the formula log2 (Control/Treatment). *indicates significance (*p* < 0.05); **indicates high significance (*p* < 0.01).

Total organic acids in the leaves of both shrubs decreased significantly with increasing water addition (*p* < 0.05). Individual organic acids contributing in the variations of total organic acids between two plant types were key metabolites in the tri-carboxylic acid (TCA) cycle and fatty acid synthesis. In *C*. *spinarum* leaves, caffeic acid, citric acid, elaidic acid, linoelaidic acid, malic acid, oleic acid, palmitic acid and sinapinic acid content was decreased (*p* < 0.05) in water treated samples (Table 1). Likewise, in *B*. *brachycarpa* leaves, a significant decrease was noted in acrylic acid, ascorbic acid, butyric acid, threonic acid, galacturonic acid, glycolic acid, lignoceric acid, oxalic acid, palmitic acid and pyruvic acid content at T1, T2 and T3 (*p* < 0.05) (Table 2). Compared to CK, the total organic acid content in the leaves of both grasses increased at T1. In *C*. *distans* leaves, the content of stearic acid, N-acetyl neuraminic acid, niacin, palmitic acid, malic acid, glycolic acid, α-linolenic acid, linoleic acid and itaconic acid increased considerably at T1, T2 and T3 (Fig. 4c). At T1, the relative content of gluconic acid, malonic acid, stearic acid, glyceric acid and propanoic acid in *A*. *sitosa* leaves was (*p* < 0.05) higher than CK (Fig. 4d). At both T2 and T3, the relative content of fumaric acid, propanoic acid, 5-O-feruloylquinic acid, glucuronic acid and niacin in *A*. *sitosa* leaves was relatively lower than CK (*p* < 0.05). Compared to the CK, a significant decrease (*p* < 0.05), in the content of quinic acid in *C*. *distans* and *A*. *sitosa* leaves was 0.73 and 2.01 folds at T2 and 1.21 and 1.49 folds at T3 respectively (Tables 3 and 4).

A total of 18 mono- and di-saccharides were identified in both shrubs (Tables 1 and 2). Compared to CK, a significant decrease was observed in galactose and rhamnose contents in *C*. *spinarum* and *B*. *brachycarpa* leaves at T1, T2 and T3 (*p* < 0.05). In *B*. *brachycarpa* leaves, total sugar content except maltose decreased at T1, T2 and T3, (Fig. 4b). For example, decrease in the relative content of turanose and glucose was 1.33 and 1.71 folds, 1.80 and 2.58 folds, 3.19 and 2.14 folds at T1, T2 and T3, respectively, as compared to CK (Table 2). Likewise, in *C*. *spinarum* leaves, lactose, mannose, galactose, maltose, tagatose and rhamnose reduced at T2 and T3 (Fig. 4a). In *C*. *distans* leaves (Fig. 4b), altrose, arabinose, fructose, glucose, β-gentiobiose, melibiose, rhamnose, erythrulose, ribose and turanose contents were higher at T1 as compared to CK; whereas, only β-gentiobiose was higher at T2 and T3 (*p* < 0.05). The relative content of β-D-glucopyranose in *A*. *sitosa* leaves improved up to 0.78 fold at T1 (*p* < 0.01). As shown in Table 4, total nine metabolites of sugar group were decreased significantly in *A*. *sitosa* leaves at T2 and T3 (*p* < 0.05). Such as, decrease in the relative content of turanose and trehalose was 2.08 and 1.82 folds at T3 as compared to CK.

Two important sugar alcohols: mannitol and myo-inositol were identified in the leaves of both shrubs (Table 1 and Table 2). Mannitol and pinitol exhibited a decreasing trend at T1 and T2 in the leaves of *C*. *spinarum* (*p* < 0.05). In addition, the relative content of myo-inositol in *C*. *spinarum* leaves increased by 0.11, 0.23 and 0.74 folds at T1, T2 and T3, respectively, as compared to CK (Table 1). Decrease in the sugar alcohol content of *B*. *brachycarpa* leaves was mainly attributed to decrease in the level of threitol and myo-inositol at T1, T2 and T3 (Fig. 4b). A total of 15 sugar alcohols were identified in grasses under different levels of watering treatment. It was noted that total relative content of sugar alcohols enhanced in *C*. *distans* leaves as a consequence of increase in the relative content of ethylene glycol, glycerol and aribitol at T1 (Fig. 4c). At T2 and T3, the relative content of mannitol, ribitol, theritol and arabitol was increased significantly compared to the CK (*p* < 0.05). In *A*. *sitosa* leaves, the relative content of arabitol increased by 0.77 fold at T2 and 0.92 fold at T3 as compared to CK (*p* < 0.05), whereas, other sugar alcohols did not show any significant change in response to water addition in *A*. *sitosa* (Table 4).

Likewise, different treatments of water also depicted noteworthy effects on the secondary metabolites in the leaves of shrubs and grasses (Fig. 4a–d). Compared to CK, a significant decline was observed for phloroglucinol, epicatechin, oleamide and 5-*p*-coumaroylquinic acid contents in *C*. *spinarum* leaves at T1, T2 and T3, respectively (*p* < 0.05), while an increasing trend was noted in the relative content of cadaverine, cerulenin, ethanolamine, 5-methyluridine and methyl galactoside at T3 (*p* < 0.05). In case of *B*. *brachycarpa* leaves, the relative content of α-tocopherol, oleamide, cerulenin, catechine, epicatechin and methyl galactoside decreased considerably at T1, T2 and T3 (*p* < 0.05), while bisabolene content was higher than CK (*p* < 0.05). Furthermore, the relative content of 2-deoxyadenosine, β-sitosterol, campesterol, magnolol, oleamide and stigmasterol increased significantly in *C*. *distans* leaves at T1 (Table 3); however no major changes were observed at T2 and T3 (*p* < 0.05). Compared to CK, the relative content of borneol and methyleugenol in *C*. *distans* leaves was increased by 4.03 fold and 1.38 fold at T2 and 3.71 fold and 1.63 fold at T3, respectively (Table 3). In *A*. *sitosa* leaves, the relative content of guanosine, oleamide, 5-methyluridine and stigmastanol was decreased considerably compared to CK at T2 and T3 (*p* < 0.05) (Fig. 4d).

### Effect of watering treatments on the lipid composition

**Figure 5:**
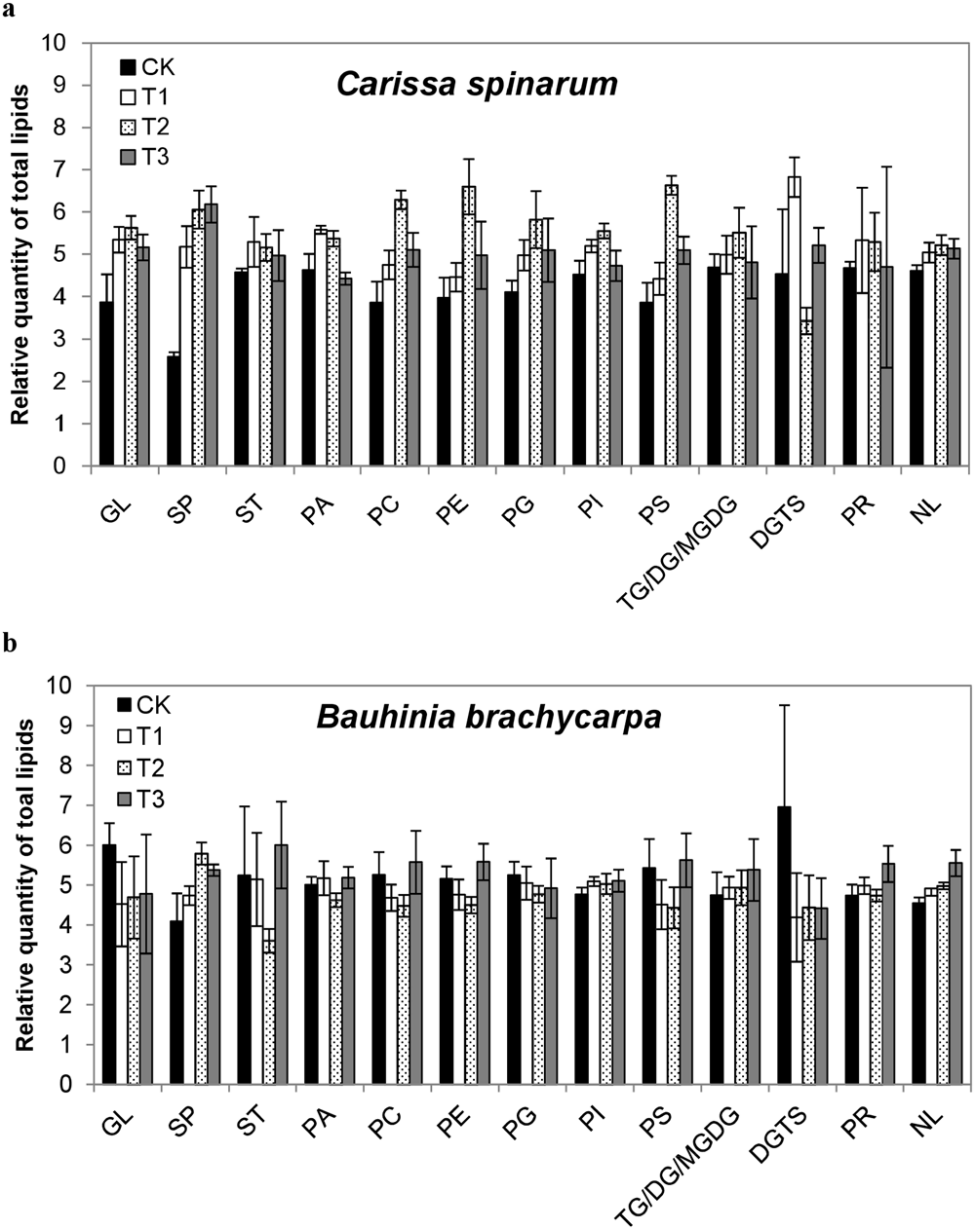
Effect of water addition on the lipid classes distribution in the leaves of two shrubs (**a**) *C*. *spinarum* (**b**) *B*. *brachycarpa*. GL, Glycerolipid; SP, Sphingolipids, ST, sterol; PC, Phosphatidylcholine; PE, Phosphatidylglycerol; PG, Phosphatidylglycerol; PI, Phosphatidylinositol; PS, Phosphatidylserine; DGTS, Diacylglyceryltrimethylhomo-Ser; PR, Prenol; TG/DG/MGDG, Trigalactosyl-/Digalactosyl-/Monogalactosyl-diacylglycerol; NL, neutral lipids + free fatty acids. Vertical bars above columns indicate standard error of each mean.

**Figure 6:**
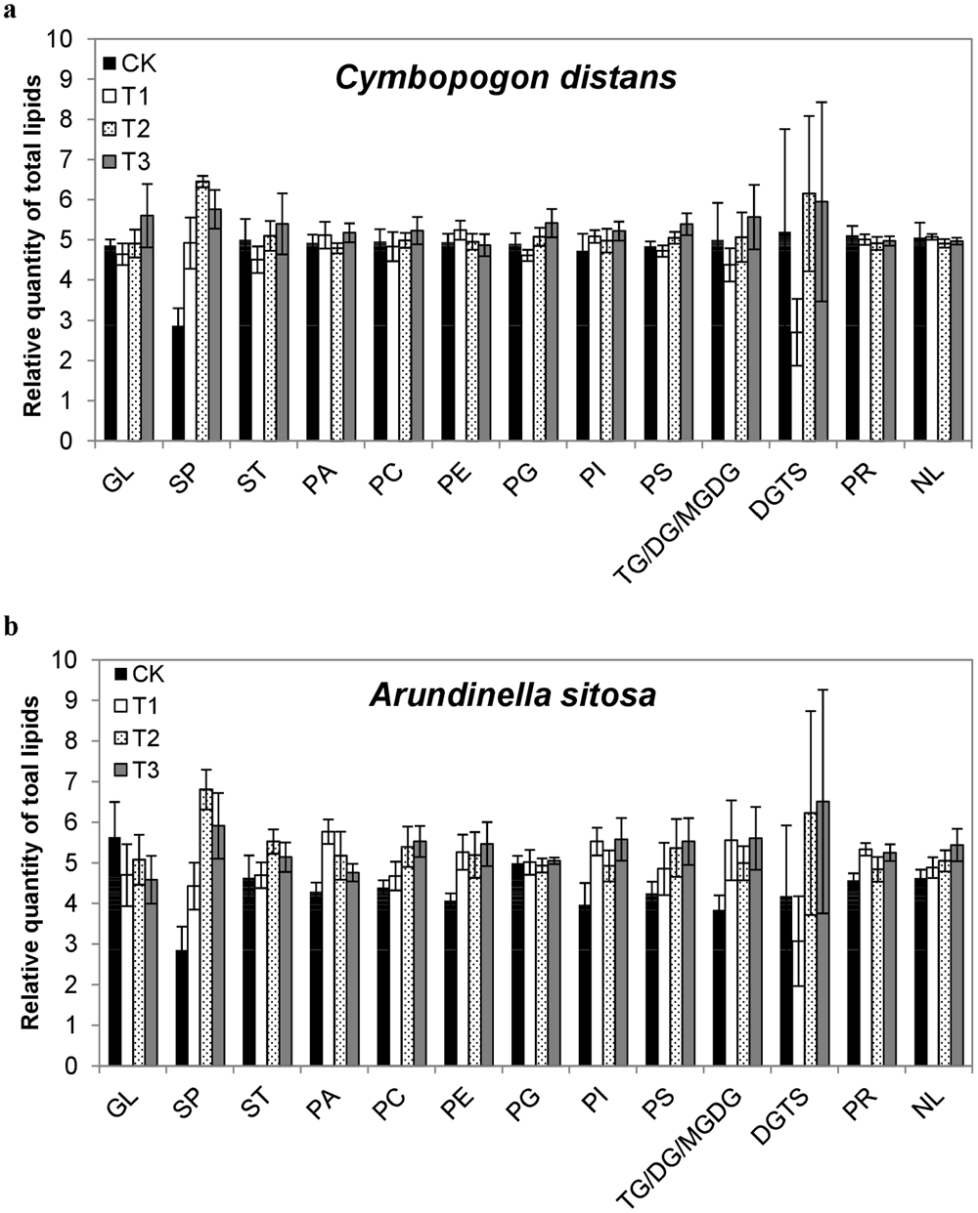
Effect of water addition on the lipid class distribution in the leaves of two grasses (**a**) *C*. *distans* and (**b**) *A*. *sitosa*. Abbreviations are as given for Fig. 5.

Like other metabolites, watering treatment depicted significant effects on the content of lipids and fatty acids in the leaves of both shrubs and grasses. As shown in Table 1, decrease in the content of total fatty acid in *C*. *spinarum* leaves was mainly due the decline in palmitic acid (C16:0), oleic acid (C18:1), elaidic acid (18:1T) and linoelaidic acid (C18:2T), at T1, T2 and T3 (*p* < 0.05). Fatty acids (FAs) are the building blocks of lipids and our results revealed that total relative lipid content of *C*. *spinarum* leaves was increased by 0.30 fold at T1, 0.41 fold at T2 and 0.26 fold at T3 (Fig. 5a). This enhancement was mainly due to increase in glycolipids (GL), sphingolipids (SP), sterol (ST), Phosphatidylcholine (PC), Phosphatidylethanolamine (PE), Phosphatidylglycerol (PG), Phosphatidylserine (PS) and neutral lipids (NL) after watering treatments (*p* < 0.05). In contrast, the relative content of palmitic acid (C16:0), linoleic acid (C18:2) and α-linolenic acid (C18:3) in *B*. *brachycarpa* leaves increased significantly at T1 (*p* < 0.05). However, lignoceric acid (C24:0) was decreased by 3.98, 2.38 and 2.81 folds at T1, T2 and T3, respectively (Table 2). Whereas, the total lipid content in *B*. *brachycarpa* leaves was decreased by 0.11 fold at T2 as compared to CK. In *C*. *distans* leaves, the relative content of palmitic acid (C16:0), stearic acid (C18:0), linoleic acid (C18:2), alpha-linolenic acid (C18:3) was improved by 1.59, 1.34, 1.52 and 1.07 folds at T2, and 1.55, 0.88, 1.47 and 1.50 folds at T3, respectively (Table 3). And only the lipid class SP of *C*. *distans* leaves was increased after water addition (Fig. 6a). In *A*. *sitosa* leaves, decline in the content of fatty acids was attributed to the decrease in myristic acid (C14:0), palmitic acid (C16:0), stearic acid (C18:0), linoleic acid (C18:2), alpha-linolenic acid (C18:3) and lignoceric acid (C24:0) at T2 and T3 (Table 4). The total lipid content was significantly increased by 0.30 fold at T2 and T3 each (Fig. 6b). The significant increase in the total lipid content in *A*. *sitosa* leaves was attributed to an increase in SP, PA, PI and PS with water addition (*p* < 0.05).

### Effects of water additions on metabolic pathways

**Figure 7:**
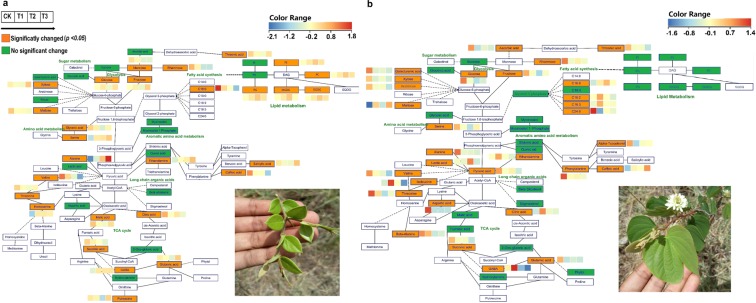
Metabolic changes involved in the primary pathways of leaves of two shrubs (**a**) *C*. *spinarum* and (**b**) *B*. *brachycarpa* under different levels of watering treatments. Metabolites, which were colored, were detected in this species. Non-colored Metabolites were not detected. Red and blue colors indicate increased and decreased metabolite concentrations, respectively. Each colored cell on the map corresponds to a normalized log response value of the metabolite with water treated samples (control, 0%; T1, +20%, T2, +40; T3, +60%) (from left to right).

**Figure 8:**
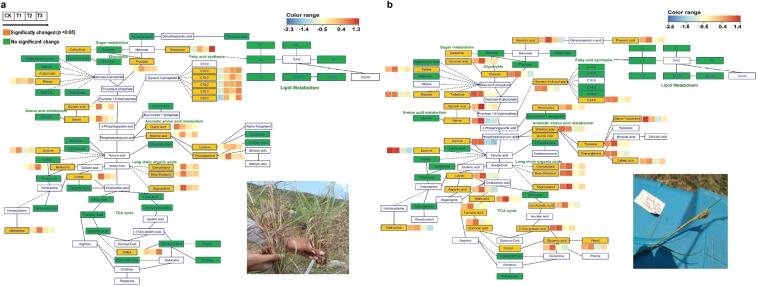
Metabolic changes involved in the primary pathways of leaves of two grass species (**a**) *C*. *distans* and (**b**) *A*. *sitosa* under different levels of watering treatments. Other information is same as given for Fig. 7.

Metabolic profiling showed that water had considerably altered the metabolic pathways of the tri-carboxylic acid cycle of respiration, glycolysis, sugar metabolism, fatty acid synthesis, lipid metabolism, shikimic acid pathway and amino acid metabolism in the leaves of plant species under investigation. As demonstrated in Fig. 7a,b, TCA cycle was affected in *C*. *spinarum* and *B*. *brachycarpa* leaves by succinic acid, malic acid and citric acid, which were substantially decreased with the addition of water (*p* < 0.05). Additionally, watering treatment has also affected the amino acid metabolism by decreasing the valine and threonine contents in *C*. *spinarum* and *B*. *brachycarpa* leaves. Whereas, in grasses, watering treatment has strongly affected the shikimate pathway by decreasing the content of quinic acid and shikimic acid (Fig. 8a,b).

## Discussions

Lipids constitute a major group of naturally occurring bio-molecules with various biological functions. Lipids are not only the structural components of cell membrane and cell wall (e.g., waxes and cutin), but also a rich source of energy that releases during the lipid metabolism^32^. They act as activators in many biochemical processes including cytoskeletal rearrangements, signal transduction, and membrane trafficking taking place in plant species^33^. And these processes are vital for cell survival, growth and differentiation, and for plant responses to environmental stresses^32^. In this study, we deliberate the role of leaf metabolites in plant species of karst areas under different levels of watering treatment.

**Figure 9:**
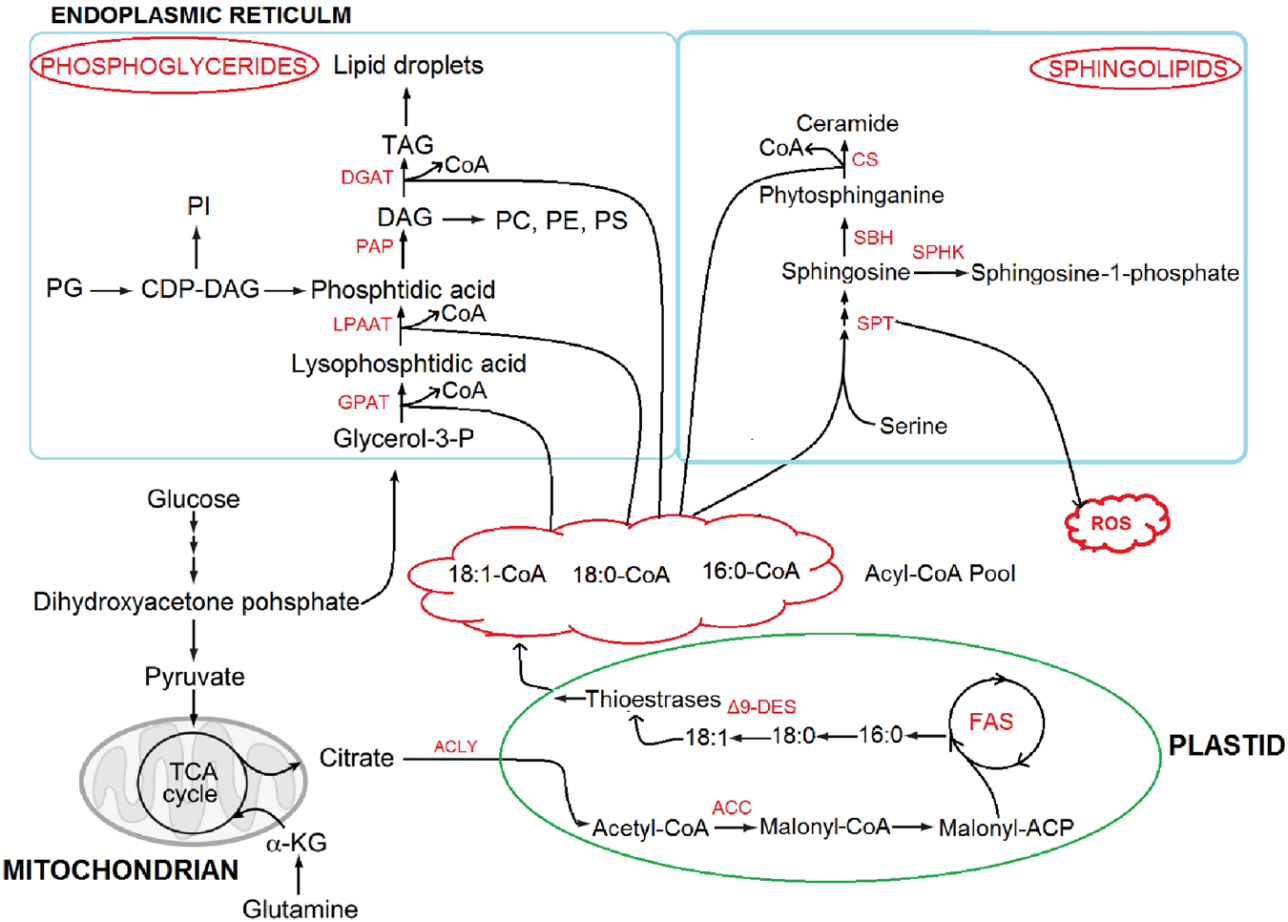
Schematic overview of the pathways involved in the synthesis of fatty acids (FAs), phosphoglycerides and sphingolipids. The enzymes involved in catalyzing steps in lipid biosynthetic pathways are indicated in red. Enzyme abbreviations: ACC, acetyl-CoA carboxylase; ACLY, ATP citrate lyase; ACC, acetyl-CoA carboxylase; CS, ceramide synthase; Δ^9^-DES, Δ^9^-desaturase; DGAT, diacylglycerol O-acyltransferase; FAS, fatty acid synthase; GPAT, glycerol 3-phosphate acyltransferase; LPAAT, lysophosphatidate acyltransferase; PAP, phosphatidate phosphohydrolase; ROS, Reactive Oxygen Species; SBK, Sphingoid base hydroxylase; SPHK, sphingosine-1-kinase; SPT, serine palmitol transferase. Metabolite abbreviations: α-KG, α-ketoglutarate; CDP-DAG, cytidine diphosphate-diacylglycerol; DAG, diacylglycerol; PA, phosphatidicacid; PC, phosphatidylcholine; PE, phosphatidylethanolamine; PG, phosphatidylglycerol; PI, phosphatidylinositol; PS, phosphatidylserine; TAG, triacylglyceride.

Sphingolipids are essential components of plant cells and contribute significantly in the growth and development in plants^34^. It has been reported that sphingolipids are directly involved in different aspects of plant development and in response of plant species to environment changes including biotic or abiotic stimuli^35^. In general, lipid content increases with increasing water and decreases in drought stress. For instance, about 24% and 31% reduction in total lipids and phospholipids in plasma membranes isolated from sunflower seedlings grown under water stress has been reported by Navari‐Izzo, *et al*.^36^. Likewise, it has been reported that water deficit caused a significant decline in the total lipid content of leaf, which rapidly increased after rehydration, showing recovery in lipid biosynthetic activities^37^. A similar trend was observed during the present investigation, whereby a significant increase in sphingolipids content was noted in the leaves of all four plant species with different treatments of water (Figs 5 and 6). According to Xu, *et al*.^38^ waterlogging causes decrease in linoleic acid (C18:2), while increase in linolenic acid (C18:3), which indicates that waterlogging might affect the production of metabolites involved in lipid biosynthesis. This advocates that additional water enhances lipid biosynthesis in the leaves of *in situ* plant species of karst areas. Firstly, during the biosynthesis of sphingolipids, sphingoid backbone is produced by the condensation of serine and palmitoyl-CoA in the presence of heterodimeric enzyme serine palmitoyltransferase (SPT) as presented in Fig. 9. This SPT enzyme is capable of generating ROS by high level of sphingoid bases^39^. Moreover, in plants lacking ORM1 or ORM2 (negative regulators of *de novo* sphingolipid synthesis), the profile of sphingolipids enhanced the production of ROS on cell wall to strengthen the plant defense mechanism against the changes in environment^40^. Secondly, flooding causes decline in the availability of oxygen for plants, which affects vital cellular and physiological processes^41^. Recently, sphingolipid profiling has been explained as a protective strategy adopted by plants under hypoxia conditions to improve tolerance to environmental stresses. A consistent increase in ceramide and hydroxyl-ceramide levels was observed in *Arabidopsis* under hypoxia conditions^42^. The production of sphingolipids with long fatty acyl moieties in *Arabidopsis* is an adoptive strategy for hypoxic tolerance^43^. However, sphingolipid signaling under wet conditions is still unresolved. Therefore, it can be concluded that increasing precipitation could be a wet stress on drought-adapted-karst plants, and the compositions of extant plant communities could be changed with the invasion of water-loving plants.

Our results also showed that the overall leaf metabolism was significantly affected by water addition (*p* < 0.05). In both shrubs, total relative contents of amino acids, organic acids, carbohydrates and other secondary metabolites were decreased consistently. However, increase in the total relative contents of metabolites in the leaves of both grasses was observed at T1; but at T2 and T3, total metabolites were decreased significantly (*p* < 0.05) in *A*. *sitosa* leaves, while no changes were observed in *C*. *distans* at the same treatments.

Amino acids function as osmolytes for regulating water content in the cells and hereafter for water-dependent processes like nutrient transport, stomatal movements and response to environmental stresses^44^. Both shrubs and grasses showed diverse metabolic behaviors in the changes of amino acid. In the present study, decrease in the content of valine and threonine in the leaves of both shrubs was observed, which was probably associated either with the inhibition of protein degradation or enhanced protein biosynthesis because plant growth was enhanced clearly with water addition^45^ and inhibited with prolonged drought stress^46^. A study conducted by Sun, *et al*.^45^ reported that isoleucine, threonine and valine were accumulated during drought and decreased after re-watering. Furthermore, changes in valine, threonine and isoleucine may be related to gluconeogenesis and relative transmission products as these amino acids are associated with pyruvate metabolism^46,47^.

Majority of the grasses are sensitive to a wide range of abiotic stresses. In the present study, an unexpected increase in the level of amino acids at T1 was observed in the leaves of both grasses, which showed their sensitivity to water. This confirms that variability in the gene pool of grasses appears to be relatively small and may provide rare chances for major steps required to tolerate such changes^48^. Likewise, some abiotic stresses, i.e. heat stress, cold acclimation, water stress, salt stress and soil nutrient deficiency, increased protein degradation and led to accumulation of NH_4_^+^ in the plant tissues^49^. The accumulation of glutamic acid in the leaves of both grasses at T1 might reflect adaptive response to generate more GABA for wet stress. Glutamic acid and GABA, which can transform from each other directly serve as nitrogen resource^50,51^. Furthermore, glutamic acid is also involved in chlorophyll biosynthesis^52,53^.

Sugar acts as an important osmolyte to stabilize membrane integrity and cell turgidity in plants^54^. Sugar activates expression of stress-linked genes^55,56^, and also functions as energy source in plants to tolerate the environmental stress^56^. Plants use sugar for growth in making cell wall fibers as energy to make other metabolites in addition to essential nutrient. Soluble sugar contributes in plant metabolism and structural growth at cellular and organ levels^57^. Our study revealed a significant decrease (*p* < 0.05) in the total sugar content of *C*. *spinarum*, *B*. *brachycarpa* and *A*. *sitosa* leaves at T2 (0.53, 0.90 and 1.88 folds) and T3 (0.32, 0.87 and 1.48 folds), respectively. These findings coincide with Sun, *et al*.^45^, who reported a significant increase in the sugar content in drought affected plants but decrease in recovering plants during the watering treatments. Additionally, galactose and rhamnose contents were also decreased in the leaves of both shrubs, and this decrease reflects changes in the structural organization of the cell wall^58^. In contrast, the sugar content such as glucose, fructose and maltose was increased in *C*. *distans* leaves at T1. It is evident that the waterlogging-tolerant plant species have more carbohydrates than the sensitive species. However, up-regulated sugar transporters, enhanced phloem loading, and more soluble sugar segregation are the important mechanisms behind the waterlog tolerance^59^.

In plants, organic acids are involved to maintain the pH level, energy metabolism and osmotic potential under various environmental stresses^60^. In *C*. *spinarum* and *B*. *brachycarpa* leaves, a significant decline was observed in the content of organic acid after watering treatments: 1.20 and 0.71 folds at T2 and 0.93 and 0.92 folds at T3, respectively, as compared to the CK (*p* < 0.05). López-Bucio, *et al*.^61^ reported a dramatic increase in the biosynthesis, accumulation and transportation of organic acids under water-stressed conditions. In this context, our results showed decreased levels of TCA metabolites such as succinic acid and malic acid in *C*. *spinarum* and *B*. *brachycarpa* leaves under well-water conditions. This confirms the hypothesis of Vanlerberghe^62^, that mitochondrial respiratory pathway such as the TCA cycle is very sensitive to abiotic stress. Waterlogging or flooding affects the activity of many enzymes i.e. aconitase, pyruvate dehydrogenase, NAD^+^-dependent isocitrate dehydrogenase, NAD^+^-dependent malate dehydrogenase, 2-oxoglutarate dehydrogenase, and NAD^+^-dependent malic enzyme, linked with TCA cycle which results in the inhibition of mitochondrial respiration^63^.

A significant decrease in the content of ascorbic acid in *C*. *spinarum* and *B*. *brachycarpa* leaves (1.60 and 1.63 folds at T2) was noted as compared to CK (Tables 1 and 2) which reflects down-regulation of the antioxidant system, amylase, and the seed germination rate^64^. These findings are comparable to Bartoli, *et al*.^65^ who reported 35% decrease in the ascorbic acid content of wheat leaves after exposure to water. Likewise, up to 30.1% decrease has been reported in the ascorbic acid of tomato plants grown under waterlogged treatment^66^. Additionally, Murshed, *et al*.^67^ reported that water stressed *Solanum lycopersicon* showed a consistent increase in the ascorbic acid content affecting the transcript levels of various antioxidant enzymes. However, ascorbic acid in leaf tissues is necessary for the regulation of the catalytic activity of ascorbate peroxidas, dehydroascorbate reductase, mono-dehydroascorbate reductase and glutathione reductase, resulting to the removal of H_2_O_2_ related molecules with the maintenance and regulation of the ascorbic acid pool^68^.

Our results clearly showed that quinic acid decreased significantly in the leaves of both grasses with water addition. These findings confirm that drought-treated plant species had higher concentrations of quinic acid^14,69^. Quinic acid is a key metabolite of the Shikimate pathway. This is an important pathway in the production of aromatic amino acids i.e. phenylalanine and tyrosine^70^, which are the precursors of flavonoids^71^. Phenolic compounds involved in altering the kinetics of peroxidation and their antioxidant function causes high reactivity as H-atom or electron donors^72^.

Organic alcohols including sugar alcohols or polyols i.e., glycol, glycerol, mannitol, myoinositol and sorbitol are often found to accumulate in many species under water-stress conditions^73^. Sugar alcohols frequently occur in many important crop plants functioning as primary photosynthetic products and playing major roles in translocation and storage^74^. A sugar alcohol, myo-inositol was differentially regulated in the leaves of both species under well-water conditions. Accumulation of myo-inositol in response to drought treatments has been reported in drought sensitive *Glycine max*^75^, *Zea mays*^45^ and *Pinus pinaster*^76^. Myo-inositol is the key metabolite and acts as a precursor of several other metabolites such as phosphatidylinositol, myo-inositol polyphosphate and a number of compatible solutes including pinitol, galactinol, raffinose-family oligosaccharides, and cell-wall polysaccharides^77^. In grasses, arabitol was found to be up-regulated with the increase of water supply. Watkinson^78^ reported that the accumulation of arabitol content in fungi played a significant role in osmotic adaptation.

Primary metabolites are involved in the synthesis of secondary metabolites such as phenolics, terpenoids, steroids and alkaloids^79^. Though, secondary metabolites are not essentially involved in the optimal growth and developmental processes in plants; but their production decreases the palatability of the plant tissues^80^. In the present study, cadavarine content was increased up to 1.16, 1.16 and 1.23 folds at T1, T2 and T3, respectively, in *C*. *spinarum* leaves (Table 1). This cadaverine compound produces as lysine catabolite and is involved in plant growth and development, cell signaling, stress response, and insect defense^81^. The α-bisabolene and β-bisabolene was accumulated in *B*. *brachycarpa* leaves during the course of water periods. Bisabolenes are present in the essential oils of a wide variety of plants but their biological role is yet unclear. The content of borneol in *C*. *distans* leaves was increased by 2.73, 4.03 and 3.71 folds at T1, T2 and T3, respectively (Table 3). Borneol is a component of camphor oil and used as a remedy for several ailments^82^. β-amyrin was accumulated in *A*. *sitosa* leaves at T1. The main function of β-amyrin is still unknown in plants, but this molecule serves as an intermediate in the synthesis of more complex tri-terpene glycosides associated with plant defense mechanism^83^.

Based on our results, watering treatments had significant effects on leaf metabolism: a significant decrease in the content of leaf metabolites in both shrubs with increasing precipitation from CK to T3, and in contrast, both of the grass species had higher leaf metabolites at T1. Firstly, the opposite responses of grasses and shrubs to variation in water supply could be associated with contrasting root distributions and competitive interactions^84^. Deep rooted shrubs could use stored water in deep soil layers^85,86^, whereas grasses have relatively shallow roots and use soil water located in upper layers of the soil^87^. Therefore, variations in the available resources may result in the competitive balance between the two different types of plants^85^. Secondly, it could also be due to the different growing pattern of grasses and shrubs in a year cycle due to variations in soil moisture content. In this study, both warm-season grasses and broadleaf shrubs initiate new growth in drought season with warming temperatures. During this period, shrubs may grow faster than grasses due to changes in soil moisture content under well-water conditions. Because, the growing point in shrubs is located at the tip of the shoots while grasses has growing point at the base^88^. However, the optimum air temperature may help to break bud dormancy in all the tea varieties^89^. He concluded that air temperature is the best treatment for breaking dormancy, enhance growth and ultimate yields of tea. In our study, the daily air temperature increased and reached a peak between 8:00 and 16:00 (Fig. S2). In Poaceae, species exhibiting summer dormancy include wild grasses such as *Poa bulbosa*^90^ and *Dactylis glomerata*^91^. These species exhibit a complete dormancy since they cease growth completely and their meristems are subjected endogenously induced dehydration even under summer irrigation^92^.

In conclusion, increasing precipitation in the karst areas of southwest China affects the leaf metabolism of plant species in this region. Based on consistent increase in the sphingolipids content in both grasses and shrubs, increasing soil moisture could be a wet-stress on these species due to their adaptation for drought conditions. Moreover, plant species under investigation have differentially responded to moisture variation in terms of metabolomics. Our results suggest that the growth of plant species might be compressed and the compositions of plant communities could be altered with changing climate in future. It can be concluded that increasing precipitation could be a wet stress on drought-adapted-karst plants, and the compositions of plant communities could be altered with the invasion of water-loving plants.

## Materials and Methods

### Description of Study area

This research is a part of the ongoing experiment I at Ecological Research Station in Jianshui County (23°59′N, 102°53′E), southeastern Yunnan, southwestern China (Fig. S3). A pot experiment was conducted in a natural grass and shrub mixed community which is located at the Karst Ecosystem Research Station. Climate of study area falls into typical subtropical monsoon type with two distinct seasons: the warm-wet season (about 85% of annual rainfall, June to October) and the dry-cool season (about 15% of annual precipitation, November to May). The long term mean annual precipitation (1981–2010) is 135 mm, with rainfall from April to May accounting for about 17.6% (http://data.cma.cn/en/?r=data/weatherBk). Mean annual air temperature of the study area is 19.8 *°*C, and mean annual precipitation is 805 mm with the lowest annual precipitation of 475 mm and the highest of 1017 mm), while average annual evaporation capacity is 2297 mm. The sunshine or radiation duration is one of the key driving forces that impact the planet's ecosystem, climate change and human activities^93^. The data from a nearby meteorological stations showed that annual average precipitation days (≥0.1 mm) are 25 during the study period. The annual average sunshine duration is 426.5 hours. The present study was done at the end of dry season, so it is likely that sunshine duration in this season was greater than wet season in all treatment plots.

In karst ecosystems, the lime soil formed from a carbonate rocks and the mean pH of the top soil is 6.29 (1 M KCL). In the study area, vegetation and soil are under serious degradation due to human disturbances which causes carbonate-rocks in the barren land. The zonal vegetation is subtropical evergreen broadleaf forests with dominant flora including *Carrisa spinarum*, *Bauhinia brachycarpa*, *Osteomeles anthyllidifolia*, *Myrsine africana*, *Dodonaea viscosa*, *Arundinella sitosa*, *Cymbopogon distans*, *Barleria cristata*, *Elsholtzia ciliate* and *Pinus yunnanensis*^94^. In the degraded areas, drought-resistant grasses and shrubs are dominant plants, e.g., perennial grasses, *C*. *distans* and *A*. *sitosa*, and two deciduous shrubs, *C*. *spinarum* and *B*. *brachycarpa*.

### Experimental design and data collection

The field experiment was based on precipitation manipulating design which consist of a randomized complete block (RCBD) with five blocks. Rainfall amounts were manipulated with appropriate methods described by Zhang, *et al*.^95^, who used the apparatus to monitoring the impact of rain redistribution on vegetation and soil traits. Totally, there were 20 plots, each 3 × 3 m in size, with five replications. Each block included four treatments of water addition, CK, T1, T2 and T3, indicating 0%, +20%, +40% and +60% relative to the monthly mean precipitation, respectively (Fig. S4). The irrigation system was installed from the beginning of April month. For each month, the monthly mean precipitation was calculated based on the climate data of 2010–2017 (Fig. S5). The natural precipitation was 143 mm at the end of dry season (from early April to late May) in 2017 (Fig. S6). Watering treatments of +20%, +40% and +60% were established by addition of rainfall and their actual manipulated precipitations were 172 mm, 200 mm and 228 mm, respectively. The plots in the control treatment (CK) received ambient levels of precipitation. In each month, the amount of irrigated water was sprayed three times to the relevant plots in the morning of every 10th day. The soil water content (SWC%) and soil pH was measured at the end of irrigation treatments (Fig. S7). The SWC% showed significant variations among precipitation treatments.

In this study, two dominant grass species (*C*. *distans and A*. *sitosa*) and two shrub species (*C*. *spinarum* and *B*. *brachycarpa*), each growing in all plots, were selected as study plants (Fig. S8). Plant leaves were sampled at the end of irrigation treatments in May (the drought season). During the sampling time, the daily air temperature increased and reached a peak between 8:00 and 16:00 (Fig. S2). The composite samples of 5 plants of each targeted species were collected from each plot. The live, green leaves were picked at the middle part of canopy from four targeted species. Leaf samples were put into liquid nitrogen tank, and transported to the laboratory with the dry-ice-box method.

### Soil water content and pH measurement

Soil pH was determined with 1:5 soil: 1 M KCl solution using a digital pH Meter (FE20/EL20; Mettler Toledo, Shanghai, China). Oven-drying method was used to measure the soil water content (SWC%). Soil samples were dried in oven at 105 °C for 48 h and SWC% was calculated as:

$$\mathrm{SWC} \% \,=\frac{{\rm{W}}1-{\rm{W}}2}{{\rm{W}}2-{\rm{W}}3}\,\times 100$$

where W1 is the weight of the container plus wet soil, W2 indicates the weight of the container plus dry soil and W3 indicates weight of the container.

### Metabolite extraction and derivation

Fresh leaf samples were collected from each plot, then immediately shifted to dry ice box and stored in laboratory at −80 °C for further studies. Metabolites profiling was achieved using Gas chromatography-mass spectrometry (GC-MS, Agilent, USA) and liquid chromatography-mass spectrometry (LC-MS). Different metabolites were extracted following the method as explained by Wu *et al*.^96^ and Du *et al*.^97^ with slight modifications. Two sets of 50-mL centrifuge tubes were tagged: set A for GC-MS analysis and set B for LC-MS analysis. Frozen leaves were lyophilized and ground to a fine powder using a ball-mill. Each tube of set A: received 100 mg of the powdered sample, whereas, 0.5 mL methanol-chloroform (3:1) and 16.6 μL 2-chloro-phenylalanine solution (3.0 mg/mL water) were added to each tube as an internal standard. This mixture was grinded at 60.0 HZ for 80 s and centrifuged at 12,000 rpm for 10 min. Next, 0.2 mL of mixture from each tube of set A was transferred to the corresponding tube of set B. Then 0.3 mL polar phase (aqueous and organic) of set A was collected independently into 1.5 mL HPLC vials and dried for 4 h in a bench top centrifugal concentrator (Labconco Corporation, Kansas City, MI). After drying the polar phase, methoximation and trimethyl silylation was carried out by incubating the dried fraction at 37 °C for 1.5 h with 80.0 μL methoximine hydrochloride (15 mg/mL in pyridine). Then, the dried fraction was incubated with 80.0 μL TMCS (BSTFA: TMCS = 99: 1) at 70 °C for 1.0 h.

### Gas chromatography-mass spectrometry (GC-MS) analysis

The derivatization of samples was carried out with a PerkinElmer gas chromatograph and Turbo Mass-Autosystem XLmass spectrometer (PerkinElmer lnc, Waltham, MS). Accurately measured 1 μL aliquot of each sample was injected into a DB-5MS capillary column (30 m × 0.25 mm × 0.25 μm) (Agilent JW Scientific, Folsom, CA). After 5 min, the GC oven temperature was adjusted at 80 °C; and after injection for 2 min, the oven temperature was raised up to 280 °C at 5 °C/min, and finally persisted at 280 °C for 27 min. The injector temperature was set at 280 °C and for the ion source at 200 °C. Helium was used as the carrier gas at a constant rate of 1.0 mL/min. Measurements were achieved with electron impact ionization (70 eV) in a full scan mode (m/z 30–550). The levels of each metabolite were identified using LECO Chroma TOF 4.3X coupled with NIST 12 library (National Institute of Standards and Technology, PerkinElmer lnc., Waltham, USA).

### UPLC-QTOF-MS/MS analysis

UPLC was performed with a Waters ACQUITY UPLC –I class system and VION IMS QTOF MASS spectrometer (Waters Corp., MA, USA) equipped with a binary pump, vacuum degasser, auto-sampler, and a column oven. Temperature of the column was maintained at 45 °C and separation was achieved on a Waters ACQUITY UPLC BEH C18 column (100 × 2.1 mm, 1.7 µm, waters). The mobile phase consisted of water and acetonitrile, both containing 0.1% (*v*/*v*) formic acid and used as mobile phases A and B, respectively. The linear gradients for UPLC-HPLC chromatographic conditions transplantation was 5% ~100% B in 15 min. The flow rate was 0.4 mL/min. The sample injection volume was 0.1~1 µL. The analysis time was 20 min.

Mass spectrometry analysis was performed on a VION IMS QTQF MASS spectrometer (Waters Corp., MA, USA) equipped with LockSpray ion source and was operated in positive and negative electrospray ionization (ESI) mode. Two independent scans with different collision energies were acquired during the running cycle: a low energy scan (CE, 4 eV) and a high energy (CE, 15–40 eV) for fragmentation. The functions settings were set at a scan time of 0.25 s and scan range 50 to 1000 amu. Argon (≥99.999%) was used as collision-induced dissociation (CID) gas. The capillary voltage and cone voltage were set at 2000 V and 40 V, respectively. The source temperature was 115 °C. The desolvation gas flow was set to 900L/h at temperature of 450 °C. Nitrogen (>99.5%) was employed as desolvation and cone gas. For lock mass correction, a 250 ng/mL standard solution of leucine-enkephalin in acetonitrile/water/formic acid (50:49.9:0.1, v/v/v) was continuously infused (5 μL/min) through the reference probe and scanned every 30 s. All data was examined using an accurate mass screening workflow within UNIFI informatics platform from Waters Corporation.

### Data analysis

GC-MS and HPLC-MS data was analyzed by Agilent's Mass Profiler Professional Software (MPP). MPP was used to exploit the high information content of MS data and determine the relationships among four watering treatment groups. We used a univariate analysis (one-way ANOVA) to test the significant differences and fold changes of the metabolites among four levels of watering treatments. As the multivariate methods take all the variables into consideration, we performed the multivariate ordination principal component analysis (PCA) to detect patterns of sample ordination in the metabolomic variables. The PC scores of the cases were subjected to one-way ANOVAs to determine the statistical differences among groups. The heat map was generated based on normalized log response value of the metabolite levels, showing significant metabolites selected by an analysis of variance (ANOVA) test using a significance level of *p* ≤ 0.05. The samples were arranged according to their watering treatments levels i.e. CK, T1, T2 and T3. The fold change of significant leaf metabolites among four groups CK, T1, T2 and T3 (cutoff of 2-fold log2, *p* < 0.05) was calculated. The pathway analysis was performed using Agilent's Mass Profiler Professional Software (MPP) for the identified significant metabolites using *Arabidopsis thaliana* pathway libraries. The wiki pathways (https://www.wikipathways.org) and Kyoto Encyclopedia of Genes and Genomes pathway database (http://www.genome.ad.jp/kegg/pathway.html) were also used for the metabolites.

For statistical analysis, we used SAS version 9.1 to perform ANOVAs and determine the effect of watering treatments and error associated with the experiment with five replications and four treatments as random effects. To identify significant differences among treatments, a mean comparison was carried out by using Duncan's multiple range with least significant difference (*p* < 0.05) test where error mean square was used to estimate the standard error (±SE) of differences between means. All graphical data analyses were performed using Microsoft Excel 2010 (Microsoft, Redmond, WA, USA), SPSS 19.0 (SPSS Inc., Chicago, IL, USA), Sigma Plot 10.0 (Systat software, Inc., 2006) and PAST 3.20^98^.

## Supplementary information

Supplementary information

## Data Availability

All analyzed data are included in this article and its Supplementary Information files.
