# Supplementary material for: Differential metabolic responses of shrubs and grasses to water additions in arid karst region, southwestern China
Source: Sci Rep. 2019 Jul 3;9:9613. doi: 10.1038/s41598-019-46083-1 (PMC6610130; doi:10.1038/s41598-019-46083-1)
Supplement: Supplementary file 1 — Supplementary information [file 41598_2019_46083_MOESM1_ESM.pdf]

Supporting Material

Differential metabolic responses of shrubs and grasses to water additions in arid karst region, southwestern China

Muhammad Umair[1], Ningxiao Sun[1], Hongmei Du[2], Jun Yuan[1], Arshad Mehmood Abbasi[3], Jiahao Wen[1], Wenjuan Yu[4], Jinxing Zhou[5], Chunjiang Liu[1,6,7]*

[1]School of Agriculture and Biology, Shanghai Jiao Tong University, Shanghai, 200240, China

[2]Design School, Shanghai Jiao Tong University, Shanghai, 200240, China

[3]Department of Environment Sciences, COMSATS University, Islamabad, Abbottabad Campus, Pakistan

[4]Instrumental Analysis Center, Shanghai Jiao Tong University, Shanghai, China

[5]Yunnan Karst Ecosystem Research Station, School of Water and Soil Conservation, Beijing Forestry University, China

[6]Shanghai Urban Forest Research Station, State Forestry Administration, China

[7]Key Laboratory of Urban Agriculture (South), Ministry of Agriculture,

22060, China

Corresponding author

Email: chjliu@sjtu.edu.cn

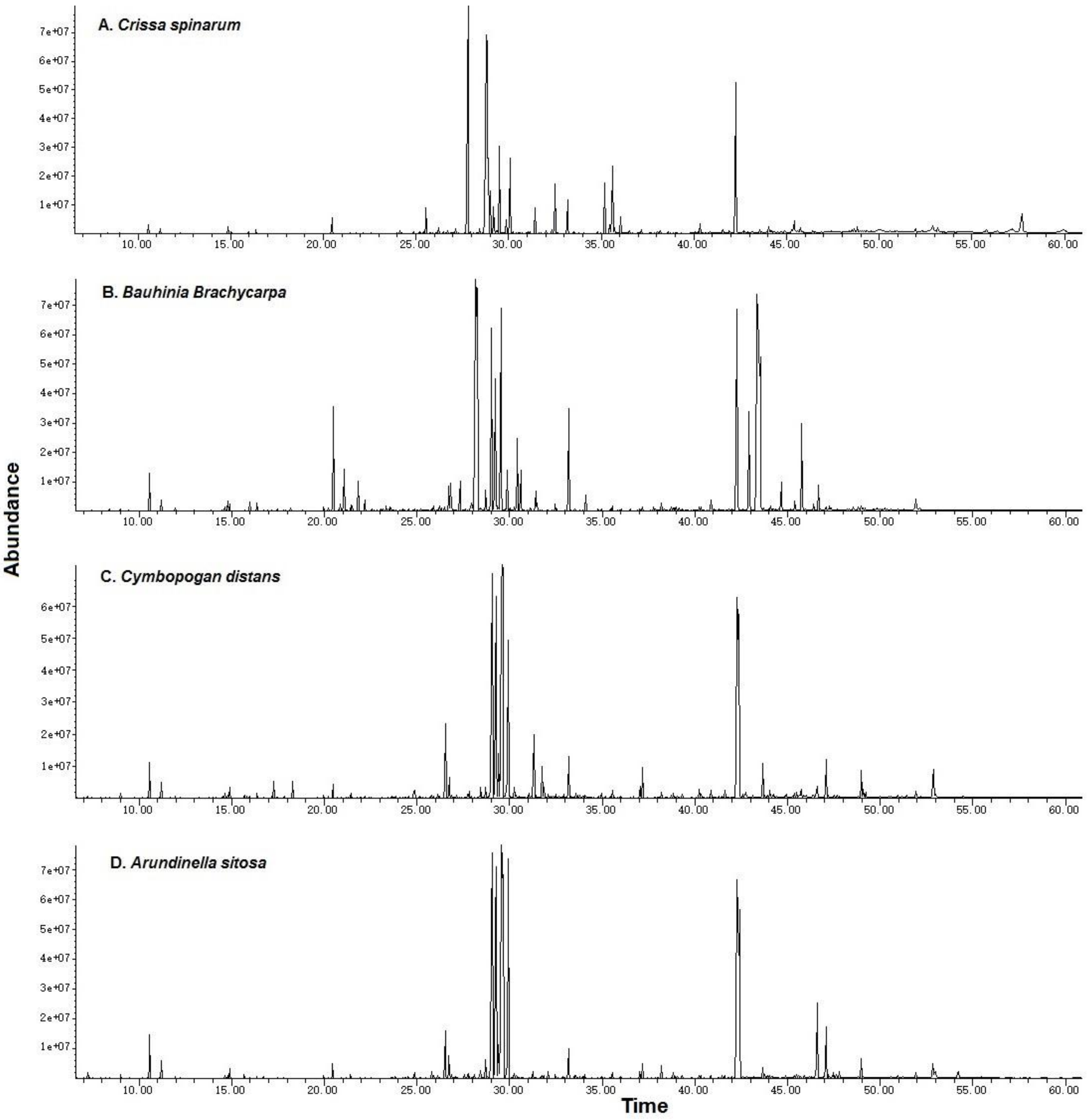

Fig. S1. A representative GC/MS chromatogram (total ion chromatogram) of TMS derivatized leaf extracts of (A) *C. spinarum* (B) *B. brachycarpa* (C) *C. distans* and (D) *A. sitosa* in response to 60% water addition.

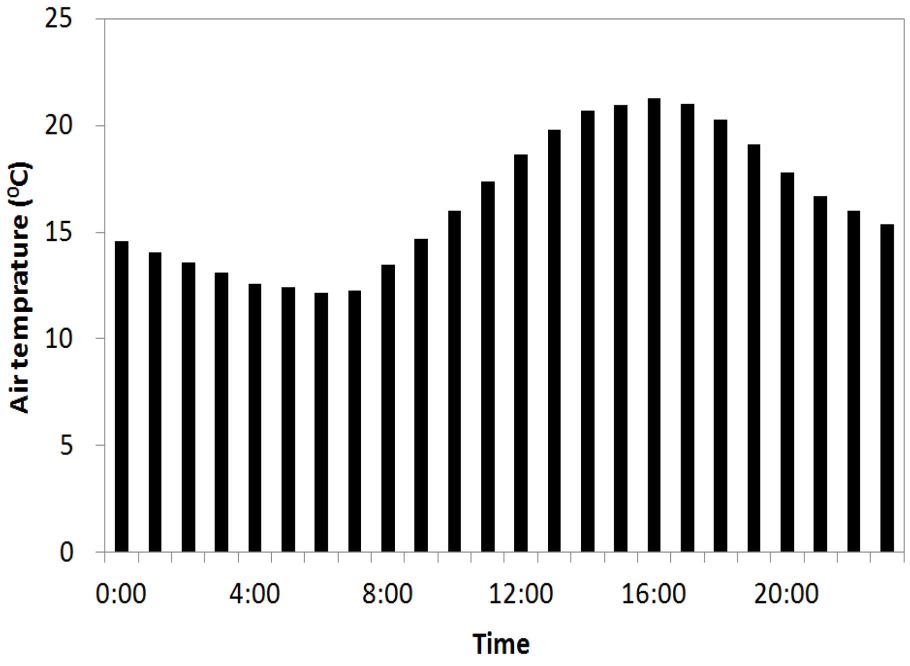

Fig. S2. Daily variation in the air temperature (^{0}C) during the study period

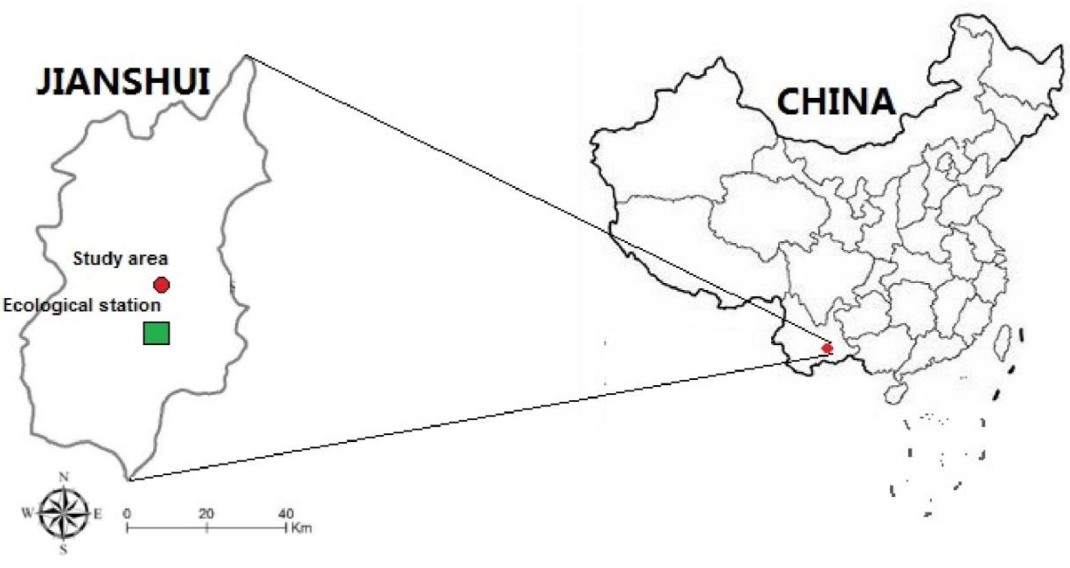

Fig. S3. Location of the study area in Jianshui county, southeastern Yunnan, southwestern, China.

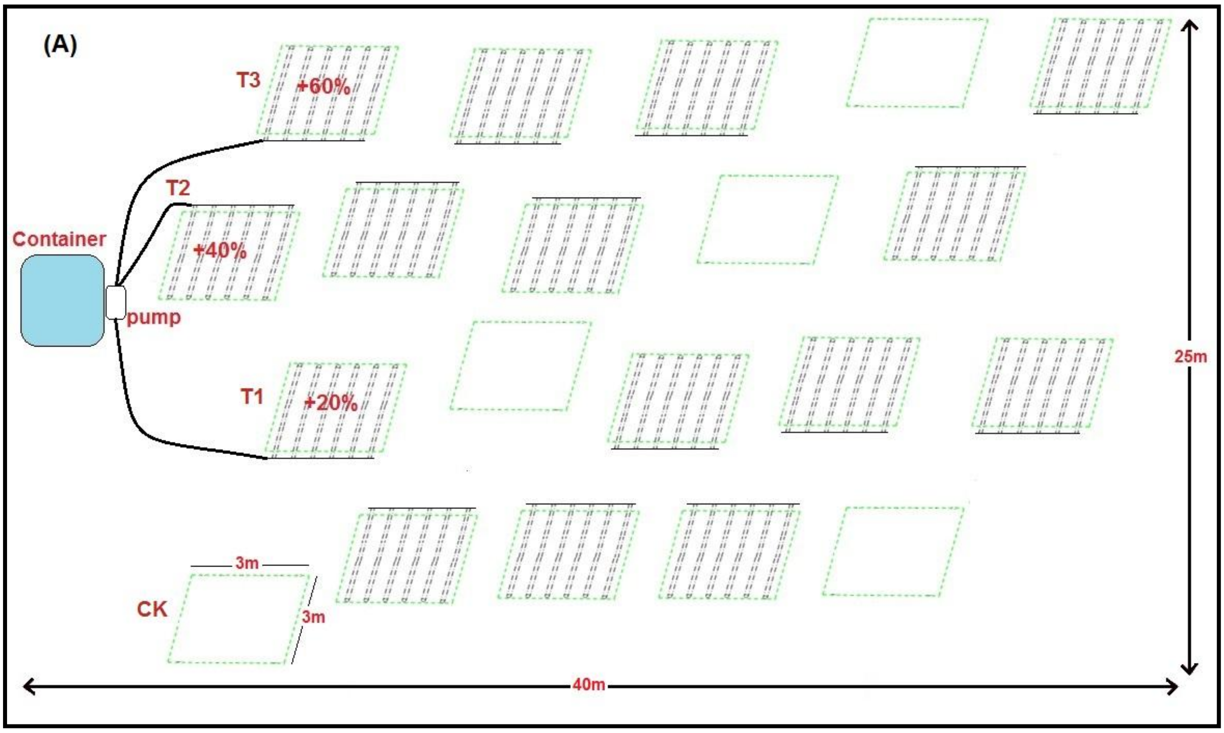

Fig. S4. The structural sketch of the experimental design installed in the arid karst area, SW China

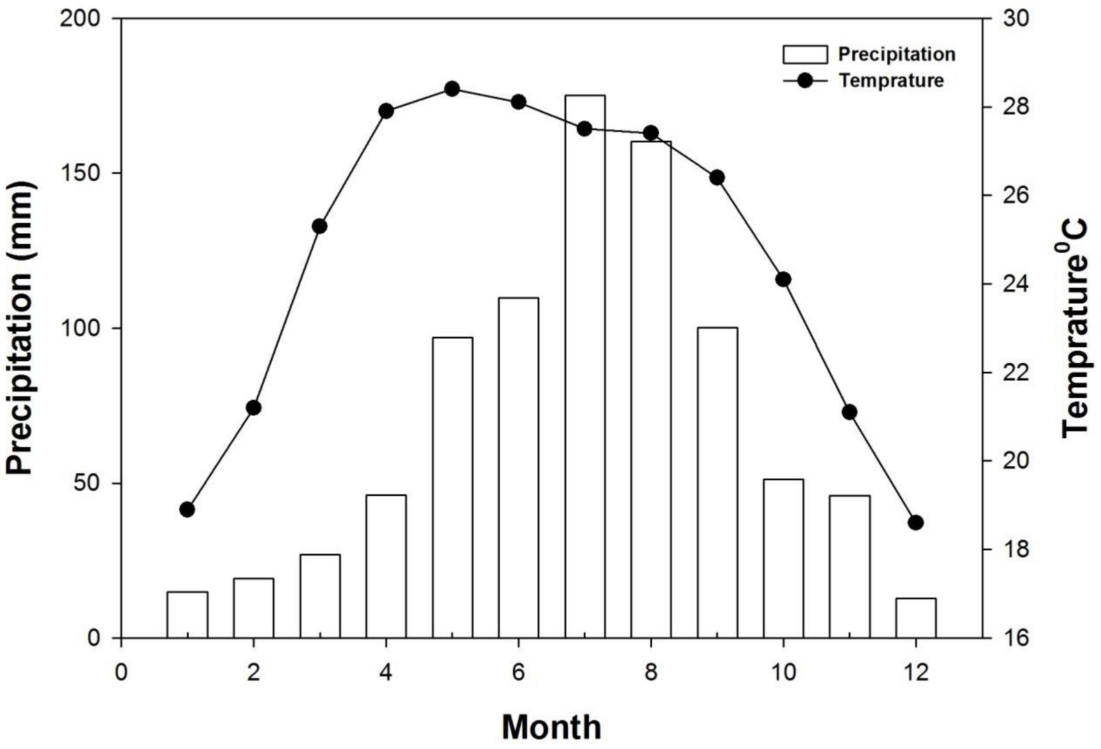

Fig. S5. The distribution of average monthly precipitation (mm) and average temperature (^{0}C) from 2010 to 2017.

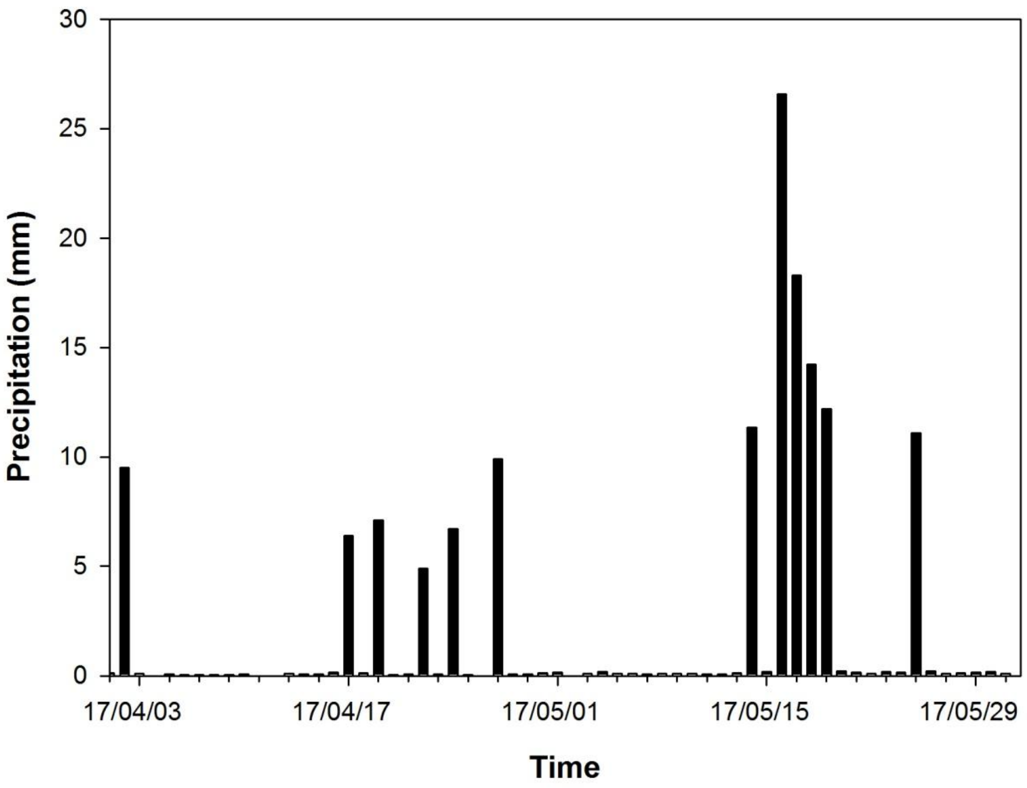

Fig. S6. Daily variation in precipitation (mm) during the study period

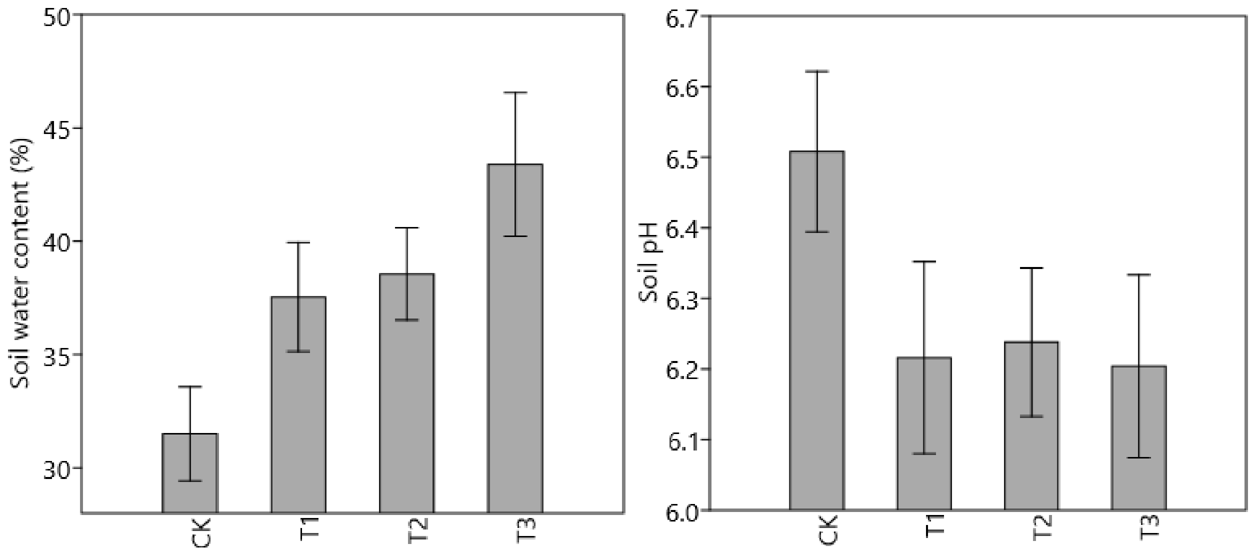

Fig. S7. Changes in soil water content (%) and soil pH in response to water addition at the sampling time.

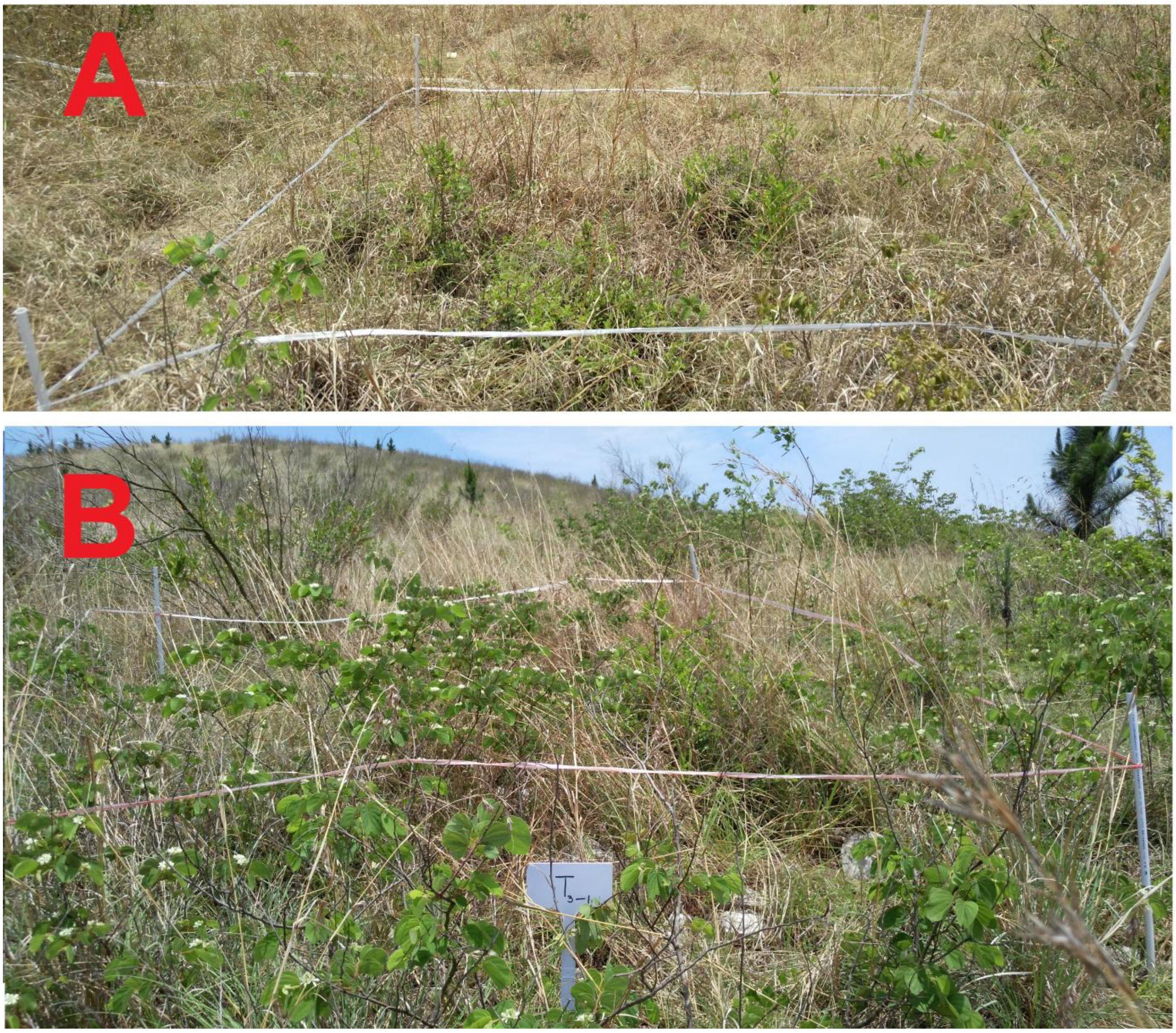

Fig. S8. The diversity of plant in the experimental plot was captured before the addition of +60% water (A) and after the addition of +60% water (B). Two dominant grass species (*C. distans* and *A. sitosa*) and two shrub species (*C. spinarum and B. brachycarpa*) are growing in this plot.

Table S1-S4 Deconvoluted total intensities (mean ± SE) of all identified metabolites in the leaves of four plant species.

Table S1

**Metabolites**	***Carrisa spinarum***			
	**CK**	**T1**	**T2**	**T3**
GABA	5633(713)b	8709(1058)a	8755(785)a	8954(1107)a
Homoserine	4029(1148)b	8663(1279)a	8486(700)a	7987(562)a
5-Oxoproline	160(26)a	233(44)a	225(53)a	198(28)a
Alanine	33(14)b	76(36)b	129(23)b	392(102)a
Aspartic acid	62(9)a	68(17)a	71(21)a	93(17)a
Glutamic acid	121(14)ab	111(20)ab	79(9)b	160(25)a
Serine	80(18)b	151(20)ab	156(21)ab	200(36)a
Threonine	97(6)a	73(11)b	62(5)b	73(4)b
Valine	142(29)a	83(24)ab	50(15)b	24(8)b
2,4,5-Trihydroxypentanoic acid	373(36)b	351(41)b	415(31)ab	534(46)a
3-hydroxy-3-methyl-glutaric acid	254(84)a	223(69)a	107(27)a	97(25)a
4-Coumaric acid	1471(176)a	1617(86)a	1510(103)a	988(133)b
4-Hydroxybenzoic acid	175(4)a	144(16)ab	118(9)b	112(22)b
5-Acetylsalicylic acid	10780(1186)ab	14733(2034)a	8404(1213)b	6480(506)b
9-Octadecenoic acid	1760(711)a	402(63)b	237(26)b	103(12)b
alpha-Oxoglutaric acid	2442(267)a	2511(575)a	2139(288)a	2446(319)a
Ascorbic acid	94(8)ab	69(25)ab	49(12)b	113(12)a
Caffeic acid	218(23)a	100(13)b	94(8)b	47(7)c
Carbamate	57(15)a	34(9)a	52(3)a	52(7)a
Chlorogenic acid	3741(525)a	3130(466)a	2542(379)a	3454(417)a
Citric acid	3179(497)a	1608(212)b	2010(226)b	1426(201)b
Galacturonic acid	3522(378)a	3466(538)a	2538(372)a	3089(383)a
Gluconic acid	236(15)a	283(64)a	248(39)a	205(31)a
Galactaric acid	1572(375)a	2347(420)a	1840(262)a	1633(148)a
Geranic acid	98(30)a	122(17)a	115(8)a	173(57)a
Glucaric acid	138(49)a	187(45)a	165(19)a	109(33)a
Glyceric acid	1765(182)a	1515(284)a	869(81)b	1308(64)ab
Lactic Acid	1363(100)ab	1722(214)a	1182(121)b	1391(101)ab
Linoelaidic acid	1494(700)a	213(30)b	128(8)b	175(60)b
Threonic acid	614(76)a	456(41)b	387(17)b	544(61)ab
Malic acid	27742(2990)a	17955(1122)bc	15273(1766)c	23554(3986)ab
Niacin	81(11)a	96(15)a	63(8)a	94(13)a
Oleic Acid	228(48)a	105(15)b	82(8)b	31(3)b
Oxalic acid	4699(519)a	3902(609)a	3962(376)a	5501(996)a
Palmitic Acid	12887(4709)a	3930(550)b	2671(278)b	1253(161)b
Propanedioic acid	380(59)a	370(49)a	276(39)a	349(79)a
Propanoic acid	75(13)a	262(130)a	111(54)a	135(18)a
Pyruvic acid	275(49)a	309(41)a	254(27)a	332(33)a
Quinic acid	270404(7059)ab	268672(33430)ab	234759(10017)b	320098(3554)a
Ribonic acid	2975(108)b	3054(356)b	2955(223)b	4123(325)a
Salicylic acid	141(11)ab	109(16)b	105(18)b	198(42)a
Sinapinic acid	7806(561)a	4338(700)b	2577(400)bc	2133(807)c

Succinic acid	4112(341)a	3391(699)ab	2670(119)ab	2336(419)b
Tartaric acid	281(32)a	197(37)ab	144(5)b	195(21)ab
Ursolic acid	73703(8109)a	60330(7944)ab	42505(2384)b	54863(7358)ab
2-alpha-Mannobiose	1688(171)a	1447(186)a	1112(101)a	1408(258)a
2-Ketoglucose	812(105)a	817(171)a	683(113)a	744(73)a
3-alpha-Mannobiose	5063(728)a	4695(888)a	5518(1057)a	4670(738)a
beta-D-Lactose	4346(1017)a	4456(613)a	3687(656)a	5363(748)a
Erythrose	15(1)a	16(3)a	14(1)a	17(1)a
Turanose	901(199)a	993(151)a	644(57)a	674(66)a
D-Allose	4256(119)a	3756(479)ab	2955(248)b	4572(310)a
Altrose	266(24)a	278(53)a	251(38)a	228(32)a
Arabinose	1845(185)a	2061(243)a	1715(143)a	2351(263)a
Fructofuranose	74(13)ab	65(13)ab	84(23)a	30(10)b
Fructose	85426(12016)ab	101921(19928)ab	71088(7353)b	122444(3701)a
Galactose	317(27)a	324(57)a	276(30)b	412(90)b
Glucose	223760(20420)ab	267154(19645)a	187847(11766)b	278325(18713)a
Lactose	6230(583)a	4334(609)b	3467(354)b	4314(824)b
Arabinose	718(103)a	648(72)a	744(138)a	988(244)a
Mannose	90(6)a	75(6)ab	58(9)b	57(9)b
D-Rhamnose	618(145)a	458(97)ab	293(22)b	552(55)ab
Ribofuranose	5424(658)a	5955(808)a	6185(418)a	4635(805)a
Tagatose	1418(221)a	1083(119)ab	1124(275)ab	551(90)b
Xylose	438(31)b	366(41)b	356(51)b	605(72)a
Floridosise	8549(1622)b	10622(2274)ab	8420(1245)b	14364(1147)a
Fucose	1759(341)a	1596(317)a	1469(397)a	1391(204)a
L-Rhamnose	1136(100)a	752(134)b	447(102)b	441(100)b
Threose	1161(139)a	1586(326)a	1394(60)a	1481(143)a
Maltose	2842(107)a	1930(270)b	1977(347)b	1688(194)b
Sucrose	101797(10715)a	114459(35395)a	131601(31410)a	122990(33317)a
Ethylene glycol	222(18)a	214(19)a	153(11)b	187(15)ab
Mannitol	4634(283)ab	2829(297)b	3710(488)b	6373(1205)a
Pinitol	182195(14662)ab	145886(27187)b	162926(8435)b	236710(21205)a
Sorbitol	380(104)a	529(105)a	325(51)a	385(50)a
Threitol	57(4)a	79(32)a	40(8)a	82(24)a
Myo-Inositol	26507(3047)b	28579(4531)b	31022(4129)b	44355(2840)a
Ribitol	447(56)a	407(45)a	357(32)a	331(8)a
Scyllo-Inositol	3802(426)a	3875(799)a	3526(378)a	4301(424)a
epicatechin	112540(20642)a	21551(8571)b	30447(9696)b	28289(11091)b
Cerulenin	8824(650)b	10105(1427)a	10355(724)a	13273(679)b
1-(ethenyloxy)-3-methylbenzene	10349(1894)a	17892(1929)b	18912(1367)b	10519(2265)b
1,3,5-Benzetriol	2207(852)a	553(80)a	390(49)a	135(28)a
1,5-Anhydrohexitol	1785(133)a	2179(260)a	2576(265)a	2113(407)a
1-Octacosanol	258(63)b	522(169)b	216(38)b	279(70)a
5-Methyluridine	3952(455)a	4181(630)ab	3385(334)b	7558(806)b
5-O-Coumaroyl-D-quinic acid	622(146)a	542(68)a	299(33)a	256(26)a
9,19-Cyclolanost-24-en-3-ol	5684(673)a	6950(1849)ab	4987(477)b	5557(1195)ab
9-Octadecenamide	223(49)a	120(49)a	39(4)a	114(29)a
Beta-Amyrin	3513(855)a	6116(1497)a	4018(520)a	4279(888)a
Beta-Sitosterol	170(9)b	166(26)a	142(11)a	151(23)a

Cadaverine	62(3)b	138(31)b	138(16)ab	160(18)a
D-Arabinono-1,4-lactone	646(62)a	732(133)a	586(24)a	639(60)a
D-Xylono-1,4-lactone	484(83)b	648(84)ab	888(60)a	520(124)b
Erythrono-1,4-lactone	376(56)a	367(63)a	338(52)a	522(141)a
Ethanolamine	762(119)b	1048(167)b	1210(210)ab	1692(159)a
Glycerol	3191(484)a	2884(275)a	2409(363)a	3479(474)a
Hydroxylamine	470(107)a	562(105)a	707(61)a	692(56)a
Methyl galactoside	12090(1715)b	10725(1488)bc	8479(381)c	19324(1109)a
Myo-Inositol 1 phosphate	383(80)a	400(58)a	290(15)a	359(99)a
Phosphoric acid monoethyl ester	281(19)a	367(74)a	264(35)a	301(38)a
Phosphoric acid trimethyl ester	11820(3440)a	11741(3235)a	12957(593)a	11566(516)a
Phytol	3194(1191)a	5360(321)a	4315(673)a	4282(352)a
Putrescine	21(3)ab	20(5)ab	10(2)b	24(4)a
Squalene	3561(1177)a	5327(1410)a	6578(1161)a	5801(2697)a

The statistically significant differences among treatments are detected by Duncan's Multiple Range Test (p <0.05).

Table S2

Metabolites	*Bauhinia brachycarpa*			
	**CK**	**T1**	**T2**	**T3**
Alanine	729(189)a	303(77)b	312(81)b	250(28)b
Isoleucine	22(7)b	53(7)a	21(4)b	13(5)b
Phenylalanine	88(14)a	40(8)b	43(11)b	36(11)b
Serine	259(30)a	164(20)b	119(24)b	188(25)b
Threonine	240(13)a	147(36)b	66(10)c	104(31)bc
Valine	242(74)a	139(34)ab	112(26)b	72(16)b
Beta-Alanine	267(43)a	253(76)b	102(14)b	117(23)b
GABA	1537(491)b	3303(361)a	675(169)b	702(292)b
5-Oxoproline	696(88)a	633(124)a	599(44)a	533(68)a
Aspartic acid	462(126)a	123(29)b	153(53)b	144(56)b
Glutamic acid	2194(161)a	1078(247)b	1179(249)b	1116(260)b
N,N-Dimethylglycine	757(238)a	153(31)b	272(105)b	54(5)b
2,4-dihydroxybutanoic acid	17(2)a	25(7)a	18(2)a	15(2)a
2-hydroxy-3-methyl-pentanoic acid	56(11)a	42(10)ab	35(8)ab	29(9)b
4-Coumaric acid	10141(1066)b	18590(4966)a	8610(1248)b	22991(4297)a
4-Hydroxybenzoic acid	176(22)a	147(25)a	156(28)a	162(25)a
Acrylic acid	95(11)a	71(9)b	52(6)c	43(6)c
Alpha-ketoglutaric acid	68(11)a	81(32)a	42(8)a	38(7)a
Alpha-Linolenic acid	588(79)b	1084(173)a	676(118)b	633(80)b
Ascorbic acid	405(56)a	166(43)b	209(49)b	176(26)b
Butyric acid	268(14)a	145(42)b	88(29)b	102(27)b
Caffeic acid	4250(811)b	9197(2178)ab	7927(1929)ab	13685(2329)a
Carbamate	61(16)a	77(13)a	65(9)a	63(10)a
Chlorogenic acid	148(30)a	121(77)a	184(53)a	163(87)a
Citric acid	69(8)b	193(38)a	99(27)b	92(25)b
Galacturonic acid	552(52)a	264(72)b	265(53)b	215(24)b
Gluconic acid	1940(453)a	1903(618)a	933(101)a	1840(824)a
Fumaric Acid	342(133)ab	465(120)a	153(14)b	225(38)ab
Galactaric acid	662(181)a	723(226)a	497(90)a	458(118)a
Glucaric acid	341(91)a	271(91)a	372(60)a	203(24)a
Glyceric acid	1078(286)a	1116(363)a	633(51)a	950(442)a
Glycolic acid	485(41)a	319(63)b	294(37)bc	254(47)c
Hydracrylic acid	123(18)a	100(20)ab	95(16)ab	76(13)b
Isoferulic acid	226(48)a	155(35)a	264(87)a	191(26)a
Itaconic acid	15(2)a	19(8)a	18(4)a	7(1)a
Lactic Acid	513(61)b	566(174)b	1135(209)a	435(92)b
Lignoceric acid	66(9)a	13(4)b	26(5)b	21(4)b
Linoleic acid	56(7)b	96(11)a	57(9)b	46(8)b
Threonic acid	1178(171)a	750(135)b	753(128)b	566(115)b
Malic acid	6326(1765)a	7835(2792)a	3867(324)a	3325(1074)a
Malonic acid	194(26)a	140(35)a	59(10)b	53(10)b
Mesoxalic acid	110(11)a	119(31)a	35(8)b	40(4)b
Methylcitric acid	1146(273)a	1334(349)a	791(171)a	750(179)a
Oxalic acid	1251(157)a	767(125)b	783(144)b	701(93)b
Palmitic Acid	1198(104)c	1926(372)b	2503(317)a	1657(206)b
Pyruvic acid	200(26)a	128(39)b	124(14)b	85(11)b
Quinic acid	172(63)a	186(67)a	128(37)a	129(51)a
Shikimic acid	1148(190)a	1091(271)a	911(199)a	912(167)a

Stearic acid	358(97)a	363(67)a	356(71)a	293(34)a
Succinic acid	765(57)a	693(126)a	527(62)b	401(73)b
Tartaric acid	1385(144)a	1424(373)a	996(119)a	1107(232)a
Vanillylmandelic acid	20(5)a	13(4)a	13(3)a	18(5)a
Floridoside	2910(512)a	2468(482)a	2506(269)a	1878(236)a
Beta-D-Lactose	1634(137)a	998(240)a	1162(293)a	1310(320)a
Cellobiose	78(8)a	55(15)a	70(17)a	68(18)a
Turanose	143(39)a	83(17)b	69(9)b	39(14)b
Allose	1312(41)a	668(90)b	858(148)b	724(128)b
Arabinose	316(19)a	253(42)b	183(23)c	187(26)c
Erythrose	42(10)a	33(7)a	48(9)a	51(11)a
Fructose	103128(16372)a	62229(11419)b	41339(9845)c	64174(15204)b
Galactose	357(38)a	243(40)b	199(38)b	189(38)b
D-Glycero-D-gulo-Heptose	162(45)a	170(40)a	142(23)a	130(16)a
Lactose	22236(2001)a	37861(18810)a	34264(6541)a	41200(18084)a
Lyxose	820(106)a	466(79)b	333(50)b	384(103)b
Rhamnose	137(17)a	72(15)b	70(15)b	77(14)b
Tagatofuranose	4179(566)b	5235(1015)ab	7915(2140)a	5682(1089)ab
Glucose	65365(8337)a	32631(7059)b	23005(5411)b	27404(6957)b
Erythrulose	335(32)a	195(29)b	181(22)b	194(27)b
L-Rhamnose	1354(253)a	629(123)b	755(141)b	581(35)b
Maltose	51438(17734)b	197247(46764)ab	101465(33943)ab	220256(71597)a
Melibiose	5998(651)a	3868(316)a	4093(918)a	3757(836)a
Sedoheptulose	899(90)a	559(157)ab	646(159)ab	486(105)b
Sucrose	6200(1757)a	5307(1186)a	6306(1097)a	6267(1412)a
Xylose	281(22)a	166(26)b	188(24)b	160(26)b
Mannitol	869(87)bc	1200(41)a	963(122)b	672(135)c
Pinitol	6386(1084)a	6738(1747)a	6699(1077)a	5123(862)a
Sorbitol	228(57)a	216(100)a	140(59)a	147(72)a
Erythritol	103(14)a	146(27)a	118(24)a	121(25)a
Threitol	268(22)a	136(39)b	172(33)b	114(22)b
Myo-Inositol	24120(2733)a	22472(4582)ab	21829(3167)ab	15983(2076)b
Ribitol	316(61)ab	465(122)a	238(23)b	284(51)ab
1,2-Ethenediol	584(93)a	539(112)a	452(86)ab	356(51)b
1,5-Anhydrohexitol	608(242)a	370(134)a	372(125)a	712(306)a
1-Heptacosanol	27(6)a	21(4)a	26(10)a	16(6)a
1-Hexacosanol	1621(272)a	1515(308)a	1203(143)a	1312(400)a
1-Octacosanol	195(29)a	182(42)ab	122(22)b	123(30)b
9,19-Cyclolanost-24-en-3-ol	134(40)a	90(27)ab	90(16)ab	62(8)b
Alpha Bisabolene	3085(926)b	10490(1208)a	4532(662)b	8814(2258)a
Alpha-Amyrin	251(71)a	113(44)a	266(101)a	254(91)a
Alpha-Tocopherol	518(78)a	190(34)b	317(77)b	305(48)b
Beta Bisabolene	2949(947)b	9846(1216)a	4703(686)b	8927(2270)a
Beta-Sitosterol	174(23)a	136(28)a	135(21)a	140(36)a
Catechine	11916(2208)a	2228(1043)b	4564(1160)b	3520(1147)b
Cerulenin	10195(960)a	7584(1423)b	5804(814)bc	5366(500)c
Epicatechin	3183(1507)a	1338(1078)ab	1922(1167)ab	506(184)b
Erythrono-1,4-lactone	350(54)a	305(91)a	284(68)a	290(107)a
Ethanolamine	1462(264)b	2467(235)a	1423(200)b	1228(194)b
Gluconolactone	102(9)a	96(23)a	91(11)a	87(13)a
Glycerol	1364(131)ab	1438(284)a	960(92)bc	879(114)c
Glycerol 3-phosphate	6880(727)a	6421(1304)ab	5687(903)ab	4597(681)b

Hydroxylamine	34(6)a	41(12)a	38(7)a	25(3)a
Inositol monophosphate	493(56)a	474(102)a	520(98)a	447(82)a
Methyl galactoside	78(10)a	36(6)b	34(8)b	40(6)b
N-Acetyl-D-glucosamine	32(4)a	25(5)a	21(5)a	30(9)a
Oleamide	175(16)a	78(22)b	83(20)b	77(12)b
Phosphoric acid monomethyl ester	21(3)a	23(7)a	21(3)a	16(3)a
Phytol	1586(206)a	1652(403)a	1417(187)a	1187(187)a
Stigmasterol	108(14)a	94(21)a	86(15)a	88(25)a
Trimethyl phosphate	5712(1173)ab	6571(1489)a	4674(616)ab	4120(776)b

The statistically significant differences among treatments are detected by Duncan's Multiple Range Test ($p < 0.05$).

Table S3

Metabolites	*Cymbopogon ditsans*			
	**CK**	**T1**	**T2**	**T3**
Glycine	513(70)b	1042(319)a	612(107)ab	409(26)b
Alanine	360(55)a	643(209)a	274(89)a	302(61)a
Isoleucine	211(31)b	275(50)b	480(33)a	235(62)b
Leucine	291(53)b	453(41)a	205(48)b	285(62)b
Lysine	150(28)b	386(72)a	224(46)b	177(27)b
Methionine	99(18)b	189(30)a	99(20)b	73(12)b
Phenylalanine	371(53)b	819(158)a	437(95)b	411(21)b
Proline	38(20)a	16(6)a	94(33)a	45(20)a
Serine	834(139)b	1646(304)a	1130(242)ab	674(182)b
Threonine	166(21)ab	316(69)a	211(51)ab	145(33)b
Tyrosine	448(69)c	692(60)ab	763(51)a	566(48)bc
Valine	577(99)a	977(194)a	722(180)a	564(34)a
Tyramine	120(26)a	217(65)a	251(79)a	177(28)a
5-Hydroxynorvaline	86(6)a	173(46)a	132(40)a	129(13)a
Beta-Alanine	5(1)b	8(1)a	5(1)ab	6(1)ab
GABA	1231(183)b	1696(228)b	1677(403)b	2752(199)a
5-Oxoproline	238(24)a	557(160)a	402(92)a	439(41)a
Aspartic acid	62(6)a	164(68)a	76(20)a	59(10)a
Glutamic acid	44(5)b	93(23)a	57(11)ab	57(4)ab
N,N-Dimethylglycine	7344(1961)b	18188(5404)a	8402(3541)b	10488(1018)ab
2-Hydroxyoctanoic acid	49(6)a	117(27)a	61(8)a	102(38)a
2-Pentenedioic acid	8(2)c	31(9)a	13(2)bc	27(2)ab
3-Heptenedioic acid	164(22)b	430(69)a	277(53)b	191(36)b
4-Coumaric acid	82(8)b	319(51)a	194(62)ab	245(100)ab
Aconitic acid	9822(3740)a	17391(4568)a	18593(6913)a	17508(688)a
Acrylic acid	180(25)a	521(249)a	530(230)a	349(85)a
Alpha-Linolenic acid	376(76)b	927(225)ab	789(153)ab	1061(160)a
Ascorbic acid	20(9)a	47(18)a	41(10)a	42(7)a
Caffeic acid	69(31)a	64(26)a	61(10)a	104(32)a
Carbamate	73(8)a	141(31)a	104(18)a	110(30)a
Citric acid	140(39)b	323(103)a	213(50)ab	197(28)ab
Fumaric acid	64(10)a	121(34)a	75(19)a	87(11)a
Galacturonic acid	803(256)a	1519(500)a	2368(957)a	1179(239)a
Galactaric acid	222(30)b	551(92)a	508(136)a	218(24)b
Gluconic acid	31(3)a	113(62)a	42(7)a	41(4)a
Glyceric acid	303(47)b	794(173)a	354(95)b	452(37)b
Glycolic acid	152(23)b	387(107)a	191(27)b	293(18)ab
Hydracrylic acid	28(5)a	54(18)a	42(7)a	50(7)a
Isoferulic acid	87(14)a	128(48)a	133(42)a	100(7)a
Itaconic acid	876(172)b	2695(1120)ab	1928(563)ab	2907(300)a
Lactic Acid	553(103)a	1164(361)a	1277(376)a	1505(283)a
Lignoceric acid	80(16)a	332(129)a	160(35)a	318(52)a
Linoleic acid	47(5)b	121(25)ab	135(35)a	130(13)a
Threonic acid	77(15)a	158(51)a	140(26)a	174(43)a
Malic acid	705(110)b	1447(319)a	1249(267)ab	1047(104)ab
Mandelic acid	151(39)a	190(34)a	162(38)a	147(15)a

Methylmaleic acid	62(8)b	99(18)b	107(24)b	168(8)a
N-Acetylneuraminic acid	187(25)b	349(9)a	243(42)ab	276(35)ab
Niacin	51(6)b	102(34)ab	114(24)a	73(6)ab
Oxalic acid	486(53)a	888(196)a	556(110)a	650(98)a
Palmitic Acid	416(44)b	1001(259)ab	1259(279)a	1219(159)a
Propanedioic acid	61(8)a	108(43)a	110(39)a	82(17)a
Propanoic acid	12(1)a	33(12)a	29(16)a	24(5)a
Quinic acid	2881(285)a	2132(386)ab	1739(571)b	1249(60)b
Sebacic acid	2859(280)b	6688(1427)a	3176(434)b	3393(581)b
Shikimic acid	1604(268)b	3021(425)a	1724(279)b	2275(190)b
Stearic acid	112(16)c	188(27)b	283(17)a	206(23)b
Succinic acid	516(94)a	957(324)a	847(206)a	870(142)a
Tricarballylic acid	438(72)a	772(170)a	663(91)a	548(73)a
3-α-Mannobiose	1442(174)a	2310(644)a	2172(401)a	3036(964)a
Alpha-D-Glucopyranose	104(3)b	192(25)a	148(30)ab	105(8)b
Beta-D-Lactose	379(99)a	1735(843)a	1132(545)a	898(310)a
Beta-D-Xylopyranose	40(3)b	72(12)a	93(13)a	95(10)a
Allose	519(99)a	882(269)a	955(251)a	795(161)a
Altrose	198(20)b	404(89)a	288(55)ab	272(39)ab
Arabinose	1315(144)b	2528(528)a	1727(463)ab	2182(126)ab
Cellobiose	577(115)a	1072(303)a	1144(351)a	969(317)a
Erythrose	13(6)a	36(12)a	29(9)a	18(2)a
Floridoside	8074(1125)a	13420(3910)a	9813(1574)a	12019(1303)a
Fructose	69346(7760)b	126623(18762)a	92172(12650)b	85255(5839)b
Galactose	318(22)b	860(232)a	537(110)ab	400(33)b
Glucose, 6-O- à-D-galactopyranosyl-	470(51)b	1340(357)a	688(83)b	890(131)ab
Lactose	1586(214)a	1823(388)a	1762(486)a	1540(405)a
Levoglucosan	10(1)b	17(2)b	29(6)a	15(2)b
Arabinose	56(4)b	115(31)a	93(17)ab	91(6)ab
Lyxose	9(2)a	15(5)a	12(5)a	10(2)a
Rhamnose	18(1)a	41(14)a	39(7)a	31(6)a
Ribofuranose	521(51)c	692(49)b	660(76)b	867(84)a
Ribo-hexos-3-ulose	66(9)a	84(28)a	70(11)a	64(9)a
Tagatofuranose	527(117)c	914(174)bc	2104(371)a	1637(217)ab
Tagatose	290(81)a	820(281)a	720(246)a	748(163)a
Talose	81437(9185)a	105957(16232)a	81204(23669)a	85307(6338)a
Trehalose	83(14)a	154(39)a	98(15)a	333(149)a
Turanose	90(17)b	266(48)a	185(37)ab	148(24)b
Glucose	12265(1227)a	17244(2673)a	16156(2095)a	16344(858)a
Hexopyranose	15(4)a	19(7)a	25(8)a	23(7)a
Lactulose	302(72)a	470(173)a	720(310)a	706(328)a
Erythrulose	120(21)b	224(50)a	118(15)b	190(36)ab
Rhamnose	460(40)c	1261(348)ab	765(131)bc	1916(234)a
Maltose	5856(1155)a	10217(2590)a	10504(2640)a	8957(2038)a
Melibiose	8826(769)b	21332(5092)a	12803(1586)b	13744(1084)b
Sucrose	80122(14977)a	139426(53168)a	147256(47272)a	114556(16874)a
Xylose	44(7)a	82(23)a	105(49)a	85(20)a
β-Gentiobiose	746(116)c	1079(147)ab	1377(194)a	1049(131)b
Arabitol	626(101)c	1143(158)b	1752(241)a	1855(169)a

Mannitol	1724(522)b	2934(458)a	1256(129)b	1227(220)b
Pinitol	25(3)a	89(44)a	23(12)a	35(11)a
Dulcitol	48(11)a	103(35)a	71(23)a	82(22)a
Ethylene glycol	304(38)b	649(159)a	472(84)ab	494(26)ab
Galactinol	9132(1110)b	34134(6425)a	17869(5268)b	19030(2344)b
Glycerol	1446(125)b	2954(713)a	2266(514)ab	2183(143)ab
Threitol	38(3)c	56(11)bc	125(36)a	112(11)ab
Myo-Inositol	4977(425)a	10081(2752)a	7772(1865)a	7482(708)a
Ribitol	49(3)b	84(17)ab	101(15)a	93(9)a
1,5-Anhydrohexitol	86(8)a	101(27)a	74(17)a	67(12)a
1-Decanol	29(7)a	28(8)a	25(6)a	37(7)a
1-Octacosanol	169(25)a	323(127)a	223(59)a	199(19)a
2-Cyclohexen-1-one	179(53)a	239(91)a	197(75)a	200(86)a
2-Deoxyadenosine	12(2)b	36(10)a	12(2)b	16(3)b
3,4-Dimethoxymandelic acid, methyl ester	152(56)a	189(54)a	872(529)a	1768(1324)a
4-Hydroxy-3-methoxyphenylglycol	517(92)a	1117(304)a	542(162)a	607(85)a
5-hydroxy-7-methoxyflavanone	268(51)a	787(208)a	549(166)a	513(120)a
5-Methyluridine	166(22)b	307(83)a	155(33)b	172(9)b
Adenine	431(76)a	772(159)a	476(183)a	789(67)a
Alpha-Terpineol	41(12)b	97(34)ab	116(34)ab	186(37)a
Arbutin	145(21)a	200(49)a	168(43)a	151(41)a
Beta-Sitosterol	559(41)b	1192(275)a	703(98)b	644(24)b
Borneol	18(8)c	118(36)bc	290(81)a	233(65)ab
Campesterol	23(2)b	59(11)a	30(6)b	35(1)b
Erythrono-1,4-lactone	19(5)a	39(12)a	30(11)a	42(14)a
Ethanolamine	1708(179)a	3701(1014)a	3001(630)a	2071(312)a
Farnesol	1012(323)b	2151(539)a	1194(344)ab	1182(358)ab
Geraniol	626(131)a	1246(539)a	1502(674)a	1634(633)a
Geranyl acetate	107(41)a	276(94)a	631(338)a	433(101)a
Guanosine	92(21)a	158(38)a	191(57)a	178(28)a
Hydroxylamine	48(6)a	73(24)a	50(10)a	34(8)a
Inosine	35(5)c	88(9)a	55(13)bc	65(12)ab
Linalool	125(48)b	105(37)b	193(61)b	418(87)a
Magnolol	179(24)b	498(191)a	163(26)b	256(40)ab
Methyl Acetopyruvate	12(3)b	26(7)ab	24(8)ab	37(6)a
Methyl galactoside	938(178)a	1945(733)a	1968(689)a	1371(155)a
Methyleugenol	3578(792)b	6481(1858)ab	9332(1901)a	9373(1239)a
N-Acetyl-D-galactosamine	189(35)b	307(41)a	164(42)b	178(26)b
N-Acetyl-D-glucosamine	7(1)c	11(1)ab	15(1)a	10(1)bc
Nerol	17(7)a	37(13)a	20(4)a	60(35)a
Oleamide	271(31)b	450(16)a	314(47)ab	369(40)ab
Phosphoric acid monoethyl ester	297(36)b	924(374)a	505(165)ab	364(27)ab
Phosphoric acid trimethyl ester	1598(142)a	3766(1312)a	3421(772)a	2756(211)a
Phytol	535(45)a	839(255)a	619(99)a	689(35)a
Stigmasterol	206(19)b	519(110)a	288(62)b	241(30)b

The statistically significant differences among treatments are detected by Duncan's Multiple Range Test ($p < 0.05$).

Table S4

Metabolites	*Arundinella sitosa*			
	**CK**	**T1**	**T2**	**T3**
Glycine	1082(107)ab	1517(433)a	535(89)b	605(153)b
Alanine	801(40)ab	1071(428)a	209(96)b	256(72)b
Isoleucine	335(82)a	433(165)a	187(46)a	220(67)a
Leucine	639(132)a	731(285)a	120(20)b	161(54)b
Lysine	268(45)ab	504(184)a	124(40)b	116(30)b
Methionine	128(27)a	133(23)a	53(7)b	57(21)b
Serine	365(109)a	553(248)a	301(98)a	228(84)a
Threonine	228(66)a	254(105)a	123(29)a	116(46)a
Tyrosine	14654(2465)ab	23681(8173)a	6428(982)b	6687(1082)b
Valine	778(127)ab	953(319)a	357(89)b	443(122)ab
Phenylalanine	660(119)a	819(235)a	254(48)b	250(57)b
5-Hydroxynorvaline	370(69)a	436(317)a	178(75)a	84(22)a
Cystathionine	72(10)ab	103(35)a	27(5)b	28(7)b
5-Oxoproline	2972(237)a	3254(966)a	989(303)b	1395(354)b
Aspartic acid	630(51)ab	941(220)a	342(84)b	358(40)b
Glutamic acid	97(20)ab	181(41)a	91(39)b	45(21)b
N,N-Dimethylglycine	107(15)b	223(74)a	75(28)b	54(16)b
β-Alanine	24749(7965)ab	37552(8969)a	11001(2676)b	21001(4043)ab
GABA	49(9)ab	64(25)a	17(1)b	20(5)b
2-Hydroxycyclohexane-1-carboxylic acid	49(20)a	39(12)a	25(7)a	26(4)a
2-Ketoglutaric acid	1743(158)a	2203(513)a	629(68)b	803(61)b
2-Methyl-4-pentenoic acid	32(3)a	40(16)a	10(2)a	22(5)a
2-Sebacic acid	1172(383)a	2061(1028)a	307(44)a	491(43)a
3-Heptenedioic acid	177(45)ab	275(100)a	39(7)b	62(3)b
3-Hydroxymandelic acid	447(62)ab	527(147)a	183(23)b	291(64)ab
4-Coumaric acid	129(32)a	160(55)a	50(10)a	74(16)a
4-Hydroxybenzoic acid	95(20)ab	119(31)a	50(7)b	67(4)ab
5-O-Feruloylquinic acid	2338(1024)a	969(257)ab	300(100)b	463(243)b
Acetopyruvic acid	46(17)ab	86(18)a	22(4)b	24(7)b
Aconitic acid (E)	9465(2108)ab	15089(4005)a	5259(1346)b	3985(606)b
Acrylic acid	515(72)ab	647(202)a	211(33)b	280(32)b
Ascorbic acid	38(11)ab	73(21)a	32(10)b	13(3)b
Beta-D-Glucopyranuronic acid	827(201)a	308(96)b	161(37)b	175(48)b
Caffeic acid	172(17)ab	274(102)a	78(18)b	135(21)ab
Carbamate	219(43)a	270(103)a	109(20)a	113(41)a
Citric acid	105(27)ab	83(28)a	42(7)b	43(15)b
Fumaric acid	192(43)ab	234(50)a	63(8)c	92(10)bc
Galacturonic acid	5290(1099)a	7993(4343)a	2207(456)a	3290(897)a
Gluconic acid	247(41)b	511(165)a	154(23)b	160(13)b
Dodecanoic acid	347(49)ab	565(173)a	160(17)b	208(15)b
Galactaric acid	1811(252)ab	3477(1094)a	879(94)b	1467(160)b
Glyceric acid	704(203)b	1465(242)a	323(62)b	477(119)b
Glycolic acid	623(137)ab	868(218)a	259(42)b	470(66)ab
Isoferulic acid	93(22)ab	123(32)a	34(7)b	43(6)b
Itaconic acid	3639(868)ab	7198(1488)a	2417(433)b	4527(1459)ab

Lactic Acid	2661(959)a	3334(225)a	2414(861)a	1424(348)a
Lignoceric acid	65(8)ab	92(37)a	23(3)b	32(4)b
Linoleic acid	30(4)ab	48(17)a	19(4)b	23(5)ab
Threonic acid	162(22)ab	251(65)a	75(5)b	99(10)b
Malic acid	8476(3561)ab	12529(3672)a	3460(973)b	2984(506)b
Methylmaleic acid	414(90)ab	686(136)a	311(48)b	405(53)ab
Myristic acid	83(11)ab	76(22)a	35(6)b	48(12)ab
Niacin	129(21)a	139(34)a	52(6)b	67(9)b
Oxalic acid	1968(245)ab	3337(1028)a	610(105)b	1143(170)b
Palmitic Acid	1694(209)a	2325(690)a	1211(242)a	1351(238)a
Propanedioic acid	94(20)b	346(134)a	62(17)b	84(26)b
Propanoic acid	222(45)b	442(89)a	122(14)b	250(60)ab
Quinic acid	49(4)a	52(11)a	12(3)b	18(4)b
Shikimic acid	2212(286)a	2245(751)a	631(134)b	935(129)b
Stearic acid	392(58)b	677(150)a	206(43)b	271(36)b
Succinic acid	1338(238)ab	1999(572)a	562(91)b	922(124)b
Vanillylmandelic acid	5826(1577)a	2432(886)b	1290(222)b	1308(410)b
α-Linolenic acid	1442(243)a	1600(516)a	904(204)a	1008(189)a
3-α-Mannobiose	2430(322)ab	2915(796)a	1155(300)b	1172(211)b
Alpha-D-fructofuranoside	496(64)ab	932(311)a	229(47)b	268(32)b
alpha-D-Glucopyranose	709(125)a	2319(1596)a	446(140)a	397(127)a
Beta-Gentiobiose	1049(142)a	1652(844)a	365(112)a	360(123)a
Altrose	1622(226)a	1680(374)a	656(111)b	831(160)ab
Cellobiose	5164(605)ab	7000(2121)a	1880(322)c	3291(545)bc
Fructose	262909(26002)ab	302405(59477)a	141883(11431)bc	112834(31260)c
Galactose	12958(1607)ab	16798(3333)a	6856(1047)b	8257(1808)b
Glucose	2329(324)a	2769(520)a	901(76)b	1011(337)b
D-Glycero-D-gulo-Heptose	243(44)a	240(109)a	86(28)a	141(48)a
Arabinose	854(99)ab	1529(546)a	421(52)b	545(52)b
Ribofuranose	279(18)a	363(115)a	179(21)a	210(23)a
Sedoheptulose	698(128)ab	840(159)a	304(42)c	343(56)bc
Tagatose	2911(90)a	3645(945)a	2172(223)a	2976(427)a
Talose	115300(10108)ab	142584(35470)a	61026(7333)b	57722(5857)b
Trehalose	1992(542)a	2065(615)a	616(123)b	565(81)b
Turanose	1009(224)a	1021(261)a	304(66)b	240(67)b
Xylofuranose	671(92)a	780(137)a	289(15)b	381(69)b
Floridoside	23378(5818)ab	26617(8754)a	8592(1544)b	12715(1422)ab
Lactulose	1841(524)a	1346(708)a	817(445)a	1107(384)a
Rhamnose	1448(252)a	1342(533)a	1153(371)a	1064(176)a
Threose	1357(171)a	1536(394)a	315(40)b	481(44)b
Maltose	6653(719)ab	7951(2240)a	2234(292)c	3214(456)bc
Melibiose	29653(8485)a	30401(10314)a	6801(1019)b	10684(802)ab
Sucrose	269261(65378)ab	391939(131516)a	140476(23942)b	127584(11852)b
Xylose	568(54)ab	869(246)a	282(23)b	374(42)b
β-Arabinopyranose	121(3)b	128(28)ab	149(14)ab	167(13)a
β-D-Glucopyranose	573(52)b	980(239)a	328(38)b	471(22)b
Arabitol	1339(100)ab	1199(247)b	1774(231)ab	1871(197)a
Glucitol	114(19)ab	144(41)a	56(11)b	77(16)ab
Mannitol	142(16)a	162(50)a	86(21)a	96(24)a

Pinitol	42(19)a	117(57)a	11(4)a	78(28)a
Dulcitol	1067(159)ab	1388(461)a	368(74)b	416(88)b
Ethylene glycol	1213(81)ab	1521(453)a	534(65)b	644(39)b
Galactinol	46654(9539)a	54238(8637)a	16298(2440)b	24110(2635)b
Glycerol	3125(590)ab	4982(1392)a	1877(415)b	1867(228)b
Arabitol	537(58)b	458(93)b	917(88)a	1016(54)a
meso-Erythritol	137(33)a	153(78)a	62(13)a	57(18)a
Myo-Inositol	11270(1429)ab	15226(3807)a	6379(1015)b	7617(1308)b
Ribitol	340(49)a	546(152)a	251(48)a	472(115)a
Scyllo-Inositol	502(44)a	671(244)a	347(87)a	300(25)a
Xylitol	226(19)a	220(38)a	275(36)a	244(65)a
1-(3-Hydroxy-4-methoxyphenyl)-1,2-ethanediol	749(227)a	1917(1198)a	313(145)a	504(234)a
1-Octacosanol	592(127)a	288(131)b	142(27)b	85(16)b
2-Deoxyadenosine	146(29)a	126(34)a	51(15)a	69(38)a
2-linoleoylglycerol	65(10)ab	81(28)a	25(5)b	25(3)b
4-Hydroxy-3-methoxyphenylglycol	443(93)a	475(288)a	97(20)a	94(32)a
5-Iodouridine	266(14)a	295(74)a	229(22)a	265(12)a
5-Methyluridine	508(98)ab	674(159)a	144(14)c	219(22)bc
Alpha-Tocopherol	115(33)a	56(15)ab	25(10)b	48(28)ab
Arbutin	29(4)ab	43(15)a	11(2)b	15(3)b
Campesterol	485(48)ab	740(260)a	188(26)b	258(15)b
Glucose, 6-O- à-D-galactopyranosyl-	417(144)ab	658(238)a	128(22)b	235(34)ab
Erythrono-1,4-lactone	425(69)a	643(273)a	191(32)a	248(50)a
Ethanolamine	3716(852)ab	6493(2060)a	2023(327)b	1788(570)b
Glycerol 3-phosphate	976(140)ab	1262(407)a	433(88)b	348(83)b
Guanosine	290(66)a	247(64)a	72(11)b	90(24)b
Hydroxylamine	114(32)a	197(99)a	68(17)a	66(24)a
Methyl galactoside	705(87)ab	958(340)a	289(45)b	415(58)ab
Myo-Inositol 1 phosphate	147(15)ab	242(77)a	99(14)b	143(17)ab
N-Acetyl glucosamine	171(30)ab	331(111)a	64(11)b	78(21)b
Oleamide	2541(366)a	3564(923)a	585(118)b	1038(97)b
Phosphate	3884(681)ab	7323(2064)a	2592(372)b	3732(527)ab
Phosphoric acid monomethyl ester	64(10)b	154(33)a	50(15)b	58(15)b
Phytol	1121(220)ab	1480(428)a	367(35)b	732(71)ab
Putrescine	936(333)a	593(134)ab	473(123)ab	252(96)b
Squalene	421(95)a	618(251)a	306(144)a	294(112)a
Stigmastanol	63(14)a	64(18)a	14(2)b	19(1)b
Stigmasterol	845(125)ab	1284(390)a	330(42)b	428(24)b
Urea	221(91)a	320(59)a	306(163)a	82(33)a
Uridine	207(16)a	246(61)a	161(18)a	206(27)a
β-Amyrin	131(34)a	942(626)a	63(31)a	64(13)a
β-Sitosterol	1882(310)ab	2492(869)a	613(79)b	825(60)b

The statistically significant differences among treatments are detected by Duncan's Multiple Range Test (p <0.05).
